# Discriminating the reaction types of plant type III polyketide synthases

**DOI:** 10.1093/bioinformatics/btx112

**Published:** 2017-03-06

**Authors:** Yugo Shimizu, Hiroyuki Ogata, Susumu Goto

**Affiliations:** Bioinformatics Center, Institute for Chemical Research, Kyoto University, Gokasho, Uji, Kyoto, Japan

## Abstract

**Motivation:**

Functional prediction of paralogs is challenging in bioinformatics because of rapid functional diversification after gene duplication events combined with parallel acquisitions of similar functions by different paralogs. Plant type III polyketide synthases (PKSs), producing various secondary metabolites, represent a paralogous family that has undergone gene duplication and functional alteration. Currently, there is no computational method available for the functional prediction of type III PKSs.

**Results:**

We developed a plant type III PKS reaction predictor, pPAP, based on the recently proposed classification of type III PKSs. pPAP combines two kinds of similarity measures: one calculated by profile hidden Markov models (pHMMs) built from functionally and structurally important partial sequence regions, and the other based on mutual information between residue positions. pPAP targets PKSs acting on ring-type starter substrates, and classifies their functions into four reaction types. The pHMM approach discriminated two reaction types with high accuracy (97.5%, 39/40), but its accuracy decreased when discriminating three reaction types (87.8%, 43/49). When combined with a correlation-based approach, all 49 PKSs were correctly discriminated, and pPAP was still highly accurate (91.4%, 64/70) even after adding other reaction types. These results suggest pPAP, which is based on linear discriminant analyses of similarity measures, is effective for plant type III PKS function prediction.

**Availability and Implementation:**

pPAP is freely available at ftp://ftp.genome.jp/pub/tools/ppap/

**Supplementary information:**

Supplementary data are available at *Bioinformatics* online.

## 1 Introduction

Gene duplication followed by mutations produces paralogous genes with related functions. Functional prediction of genes in a paralogous gene family is challenging in bioinformatics because of rapid functional diversification after gene duplication and occasional independent acquisition of similar functions in different paralogous lineages (Pichersky and Lewinsohn, 2011; Zallot *et al.*, 2016). Plant type III polyketide synthases (PKSs) are one such paralogous family and produce polyketides (PKs), a group of secondary metabolites exhibiting large diversity in their chemical structures and physiological functions (Abe and Morita, 2010). PKSs are classified into three types (I, II and III) based on their domain structure and subunit composition (Austin and Noel, 2003). Type I and II PKSs form multi-domain-containing complexes, whereas type III PKSs have a single ketosynthase domain and form homodimers (80–90 kDa).

**Fig. 1:**
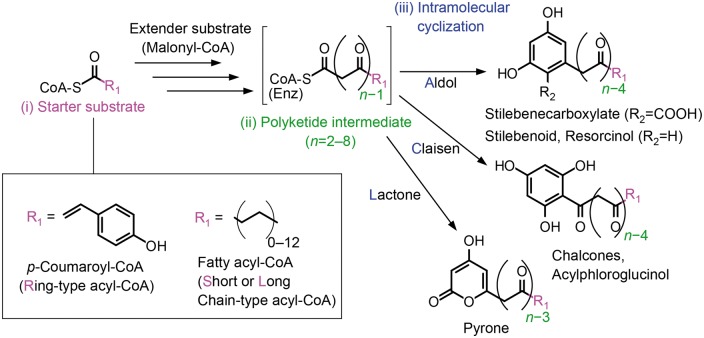
Typical reaction scheme of type III PKSs. Three features, (i) variation in the acyl part (‘R_1_’) of the starter substrate, (ii) the length of the intermediate and (iii) the mechanism of intramolecular cyclization, are used to define reaction types (e.g. R-3m-L, Sb-4-C and L-5-A). Each reaction type is represented by three elements corresponding to each of the three features. (i) The first element (e.g. R, Sb or L) represents the structure of the acyl part: ring, R; short chain (C_2_ to C_12_), S; long chain (up to C_26_), L. Additional characters are used for specific acyl-groups: branched chain, b; carboxyl, c; hydroxyl, h; nitrogen, n. (ii) The second element (e.g. 3m, 4 or 5) represents the number of methylenecarbonyl units in the intermediate, indicated by ‘*n*’, and unusual extender substrates other than malonyl-CoA: methylmalonyl-CoA, m; ethylmalonyl-CoA, e; acetoacetyl-CoA, a; diketide-CoA, d. (iii) The third element (e.g. L, C or A) represents the mechanism of intramolecular cyclization: lactone, L; Claisen, C; aldol, A; no cyclization, X; nitrogen–carbon, n; miscellaneous, +

Type III PKSs accept various acyl-CoAs called starter substrates and catalyze a cycle of decarboxylative condensations between the starter substrate and malonyl-CoA extender units to produce a polyketide intermediate. Then, the intermediate undergoes intramolecular cyclization to yield a PK product. Despite the simple structure of type III PKSs, variations in starter substrates, numbers of extensions (and rare extender substrates) and reaction mechanisms of intramolecular cyclization generate a large diversity of PK products (Fig. 1). Type I and II PKSs have been classified based on their domain organizations, which has helped predict PK products (Hertweck, 2009; Medema *et al.*, 2011). However, due to their structural simplicity, an appropriate classification system has only recently been established for type III PKSs, where their reactions were classified by a combination of three features: (i) the starter substrate, (ii) extended structure of the intermediate and (iii) intramolecular cyclization, which are connected by hyphens to form a ‘reaction type’ identifier (Shimizu *et al.*, 2017).

Type III PKSs from plants have an especially wide variety of starter substrates and reaction types compared with those from bacteria and other eukaryotes. Chalcone synthases (CHSs) mainly use *p*-coumaroyl-CoA as a starter substrate and perform three condensations with malonyl-CoA followed by intramolecular Claisen cyclization (R-4-C type; R for Ring-type starter, 4 for four keto groups in the linear intermediate, C for Claisen cyclization), which produces naringenin chalcone—a precursor of the flavonoid pathway (Austin and Noel, 2003). Reflecting the important functions of flavonoid compounds in protecting against UV damage, microbial infection and animal feeding, CHS is prevalent among plants and its protein sequence is highly conserved (Harborne and Williams, 2000). Non-CHS type III PKSs are found in limited families, genera or species and are assumed to have gained their functions via independent gene duplication events (Jiang *et al.*, 2008). This evolutionary scenario makes it difficult to predict their reactions based on simple homology searches and phylogenetic trees (Weng and Noel, 2012), except for alkylresorcylic acid synthase/alkylresorcinol synthase (ARAS/ARS; L-4-A) and anther-specific chalcone synthase-like enzyme (ASCL; Lh-4-L), which are only found in specific clades and are easily discriminated on the tree (Supplementary Fig. S1).

To discover candidates for new secondary metabolite enzymes in genomes, three computational tools have been developed to detect type III PKSs from DNA or protein sequences using different methods: (i) NRPS-PKS (Ansari *et al.*, 2004) uses pairwise sequence comparisons with template domains extracted from experimentally characterized PKS sequences; (ii) Antibiotics and Secondary Metabolite Analysis SHell uses profile hidden Markov models (pHMMs) (Medema *et al.*, 2011); and (iii) PKSIIIexplorer employs a machine learning method using a transductive support vector machine, trained by dipeptide and multiple peptide frequencies (Vijayan *et al.*, 2011). However, these tools only predict whether the query sequence is type III and provide no detailed information about its substrate or reaction type.

Here, we propose a new method to predict the reaction types of plant type III PKSs that mainly use ring acyl-CoA or short chain acyl-CoA as starter substrates. Because phylogenetic analyses of plant type III PKSs have shown that functional prediction based only on sequence similarity is difficult (Supplementary Fig. S1) (Shimizu *et al.*, 2017), we focused on the protein segments that exhibit 3D structural specificity for the reaction types R-4-C and R-4-A (Austin *et al.*, 2004). We also considered correlations of residue pairs. In the evolutionary history of a protein family, functionally similar proteins maintain environments to keep the function from mutations. After one residue is mutated, another mutation that represses the effect of the first mutation may occur to preserve the function, which is known as a compensatory mutation (Göbel *et al.*, 1994). We used correlated mutation analysis (CMA) based on mutual information to detect co-evolved pairs that may be functionally or structurally important (Dunn *et al.*, 2008). A pipeline that implemented a linear discriminant analysis combining pHMMs of the extracted segments and correlation scores by CMA discriminated R-4-A, R-4-C, R-2-X and other types with high accuracy.

## 2 Materials and methods

### 2.1 Sequence data

Amino acid sequences of plant type III PKSs with experimentally characterized reactions were retrieved from the GenBank database (Clark *et al.*, 2016). Among the 111 sequences obtained, 82 representative PKS sequences were selected by keeping the sequence identity below 90% between sequences belonging to the same reaction type using the CD-HIT program (Fu *et al.*, 2012). They consisted of 13 R-4-A, 27 R-4-C, nine R-2-X and 33 other type (four Rn-2-n/Rn-4-Cn, two R-4-C R-2-X bifunctional, three R-*-L, 12 S-*-*, six L-*-A and six Lh-4-L, where ‘*’ means any possible element) PKSs. The reaction types were assigned based on the main or representative reactions. In this research, R-2d-X was included in R-2-X. The representative PKSs, their GenBank accessions and reaction types are listed in Supplementary Table S1.

### 2.2 Multiple sequence alignments and phylogenetic trees

Multiple sequence alignment (MSA) of protein sequences for each or all of the four reaction types was performed by MAFFT 7 (Katoh and Standley, 2013) with the L-INS-i option. Alignment between a query sequence and an existing alignment was performed by MAFFT with the –add option. A phylogenetic tree of representative plant type III PKSs (Supplementary Fig. S1) was constructed by the maximum-likelihood method in FastTree (Price *et al.*, 2010), with highly gapped positions trimmed by trimAl (Capella-Gutiérrez *et al.*, 2009) and a bacterial type III PKS, *Streptomyces griseus* RppA, used as the outgroup.

### 2.3 Profile hidden Markov models

The amino acid positions in *Medicago sativa* CHS2 (R-4-C) exhibiting different 3D structure from *Pinus sylvestris* STS (R-4-A) were retrieved as described in Austin *et al.* (2004). These positions consisted of four discrete segments called Areas 1, 2, 3 and 4. The residue positions included in these Areas were obtained from the MSA. pHMMs of combinations of these Areas were constructed by HMMER 3 (Eddy, 2011) after concatenating them. The reaction types were predicted by the pHMM that exhibited the highest score among the pHMMs and the accuracy of the predictions was tested by leave-one-out cross-validation (LOOCV) and repeated random sub-sampling validation (RRSV). In the RRSV, 50% (the decimal was rounded up) of a dataset for one reaction type were randomly picked up as a training set and the other 50% were used as the test set. This process was repeated 10 times for each dataset and their average accuracy was calculated.

To determine the feasibility of reaction type prediction using HMM scores, principal component analysis (PCA) on standardized HMM scores was performed using the prcomp function of R (R Core Team, 2016); the inputs were three dimensional vectors consisting of HMM scores for the three reaction types. The HMM scores were calculated by the leave-one-out method where the score of a test set sequence was calculated by pHMMs constructed from a training set composed of sequences excluding the test set. If the HMM score of a query was less than zero or ‘no score’, which means no significant match was detected by the pHMM, the score was set to zero.

### 2.4 Correlated mutation analysis

#### 2.4.1 Selection of highly correlated residue-pairs

Mutual information (*MI*) is a measure of shared information between two variables. *MI* as a measure of correlated residue pairs of two positions *i* and *j* in a MSA is defined as:

$$MI\left(i,j\right)=\sum_{X\in {AA}_{i}}\sum_{Y\in {AA}_{j}}p_{i,j}\left(X,Y\right)\log\frac{p_{i,j}\left(X,Y\right)}{p_{i}\left(X\right)p_{j}\left(Y\right)}$$

where *AA_i_* is a set of amino acids and a gap observed at *i*, *p_i_*(*X*) is the frequency of an amino acid *X* appearing at *i*, *p_i, j_*(*X*, *Y*) is the joint frequency of amino acids *X* and *Y* appearing at *i* and *j*, respectively. Because *MI* requires hundreds of sequences to detect a correlation signal and does not filter out background noise such as an organism's phylogeny (Martin *et al.*, 2005), a corrected version of *MI* called *MIp* was developed (Dunn *et al.*, 2008). The *MIp* value is calculated by the following formulas:

$$MIp\left(i,j\right)=MI\left(i,j\right)-APC(i,j)$$

$$APC\left(i,j\right)=\frac{MI\left(i,\bar{x}\right)MI\left(j,\bar{x}\right)}{\bar{MI}}$$

$$MI\left(i,\bar{x}\right)=\frac{1}{n-1}\sum_{x\neq i}MI(i,x)$$

$$\bar{MI}=\frac{2}{n(n-1)}\sum_{x<y}MI(x,y)$$

where *APC* is the average product correction that corresponds to a large part of the background noise and phylogenetic effects.

We used the *MIp* value to detect highly correlated position pairs among the PKS sequences of a reaction type. These pairs were extracted from all the combinations of position pairs by the threshold: Z-score > 3.0. Here, the Z-score was calculated by the formula:

$$Z_{MIp}\left(i,j\right)=\frac{MIp\left(i,j\right)-\mu }{SD}$$

where *μ* and *SD* are the mean and standard deviation of *MIp*(*i*, *j*) in all possible position pairs (*i*, *j*), respectively. Gapped positions at both termini and highly gapped (>80%) positions in internal regions were manually removed.

#### 2.4.2 Correlation scores

Using the MSA of a reaction type, we calculated the residue frequency *p_i_* for position *i* and residue-pair frequency *p_ij_* for each position pair (*i*, *j*) to construct a correlation model of the reaction type. To measure the compatibility of a query sequence with the correlation model, we calculated the probability that each residue pair (*X_q_*, *Y_q_*) of the query was randomly generated by each residue *X_q_* and *Y_q_* under the condition of the reaction type. Then, we summed the log probabilities for highly correlated position pairs among the proteins of the reaction type to calculate the correlation score *S_cor_*:

$$S_{cor}=\frac{1}{C}\sum_{\left(i,\;j\right)\in A}\log\frac{p_{i,j}\left(X_{q},Y_{q}\right)}{p_{i}\left(X_{q}\right)p_{j}\left(Y_{q}\right)}$$

Here, *A* is a set of highly correlated position pairs (*i*, *j*) (*i *<* j*) defined in 2.4.1 and the corresponding positions in the query sequence were determined by aligning it with the existing alignment of the reaction type. *C* is the number of position pairs (*i*, *j*) in *A*. This constant is used to standardize different numbers of highly correlated position pairs from each reaction type.

To avoid the zero-frequency problem caused by an absence of residues in a position in the training sets, we used a pseudocount for the non-existent residues. Here, we used a simple additive smoothing method with the parameters *α* and *β* to calculate the smoothed frequencies *p_i_*(*X*) and *p_i_*_,__*j*_(*X*, *Y*) as:

$$p_{i}\left(X\right)=\frac{n_{i}\left(X\right)+\alpha }{N+\alpha d_{s}}$$

$$p_{i,j}\left(X,Y\right)=\frac{n_{i,j}\left(X,Y\right)+\beta }{N+\beta d_{p}}$$

where *n_i_*(*X*) and *n_i_*_,__*j*_(*X*, *Y*) are the numbers of residue *X* and residue pair (*X*, *Y*) observed in the MSA at position *i* and position pair (*i*, *j*), respectively, *N* is the number of sequences used for training, and *d_s_* and *d_p_* are the numbers of possible arguments (i.e., *d_s_* = 21 and *d_p_* = 21^2^). The value of *α* was set to 1/21. *β* was calculated by the formula:

$$\beta =\alpha^{2}/\left(N+2\alpha d_{s}\right)$$

derived from the assumption:

$$n_{i,j}\left(X,Y\right)=n_{i}\left(X\right)=n_{j}\left(Y\right)=0\;\Rightarrow \frac{p_{i,j}\left(X,Y\right)}{p_{i}\left(X\right)p_{j}\left(Y\right)}=1$$

where *β* is adjusted so that a non-observed pair $\left(X,Y\right)$ composed of non-observed residues *X* and *Y* does not affect the score. To determine the feasibility of predicting reaction types using HMM scores and correlation scores, PCA was performed as described in 2.3, but the input vectors here consisted of six scores.

### 2.5 Linear discriminant analysis

The discrimination of reaction types was performed by linear discriminant analysis (LDA) using the lda function in the MASS library of R (Venables and Ripley, 2002). LDA estimates the discriminant hyperplane represented by a linear combination of the features and maximally separates a training set into two or more labeled groups. The classifiers of three reaction types, R-4-A, R-4-C and R-2-X, were defined by the hyperplanes estimated from standardized HMM and correlation scores based on the whole set of sequences. A combined classifier was constructed by successively applying the three classifiers and named as pPAP, for plant PKS Analysis and Prediction (see Section 3.5). The power of discrimination for each classifier and the combined classifier was tested based on the scores derived from the HMM and correlation models, from which the sequence under test was excluded (akin to the leave-one-out method). pPAP was applied to the prediction of reaction types of 636 plant type III PKS candidates extracted from the KEGG GENES database (Kanehisa *et al.*, 2016) (Supplementary Data).

## 3 Results

### 3.1 Area selection and MSA

**Fig. 2:**
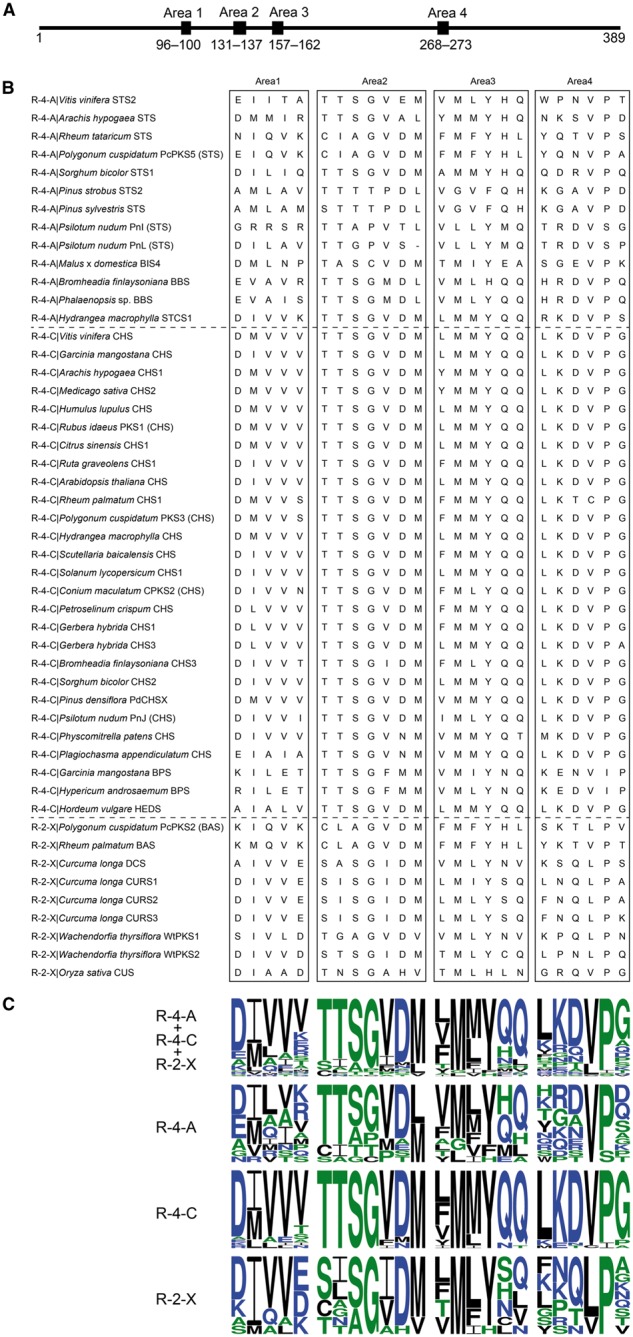
(**A**) Positions of Areas 1–4 in the whole protein sequence of *M. sativa* CHS2. (**B**) Area 1–4 parts in the MSA of known R-4-A-, R-4-C- and R-2-X-type plant type III PKSs. (**C**) Sequence logos for each reaction type and all three types together. Hydrophilic, neutral and hydrophobic residues are blue (DEKNQR), green (AGHPST), and black (CFILMVWY), respectively. The conservation of residues in MSAs was represented by sequence logos using WebLogo3 (http://weblogo.threeplusone.com/) (Color version of this figure is available at *Bioinformatics* online.)

We extracted sequences corresponding to Areas 1–4 in known plant type III PKSs of the R-4-A, R-4-C and R-2-X types from their MSAs using *M*. *sativa* CHS2 as a reference (Fig. 2A and B). Figure 2C shows the variation of residues at each position in the Areas. The residues in all Areas were well conserved for the R-4-C type, especially in Areas 2 (TTSGVDM) and 4 (LKDVPG). The residues in Area 1 and Area 2 were modestly conserved in the R-2-X and R-4-A types, respectively. The frequently appearing residues in these areas were similar to those of the R-4-C type. In the other combinations of Areas and reaction types, the residues were comparatively variable.

### 3.2 HMM scores

In this study, we constructed HMM profiles from partial sequences of the R-4-A, R-4-C and R-2-X types and from whole sequences of these types for comparison. The other reaction types such as R-4-L and S-6-AL were not used for pHMM construction because there are few known sequences of these types. All possible combinations of Areas 1–4 were used as partial sequences to construct pHMMs. The accuracy of reaction type prediction using the HMM scores was tested as follows.

First, we used a dataset that consisted of 13 R-4-A and 27 R-4-C sequences to assess the effectiveness of discrimination using the 3D structural information for pHMMs. As a result of LOOCV, the pHMMs using Area 1 + 3 + 4 and Area 1 + 4 showed the highest accuracy (39/40; 97.5%) in discriminating the two types (Supplementary Table S2). The accuracy of pHMMs using all Areas (i.e., Area 1 + 2 + 3 + 4) or the whole sequence was 36/40 (90.0%). Most pHMMs failed to correctly predict stilbenecarboxylate synthase (STCS; R-4-A) of *Hydrangea macrophylla*, probably because there were no similar sequences in the dataset. We also tested this HMM-based prediction by RRSV (Supplementary Table S3). Although the accuracies by RRSV were a bit lower than those by LOOCV in most combinations of Areas, some combinations still exhibited high accuracy (e.g. pHMMs using Area 1 + 3 + 4 and Area 3 + 4 showed the highest accuracy: 90.0% for R-4-A, 96.9% for R-4-C, 94.7% in total).

**Fig. 3:**
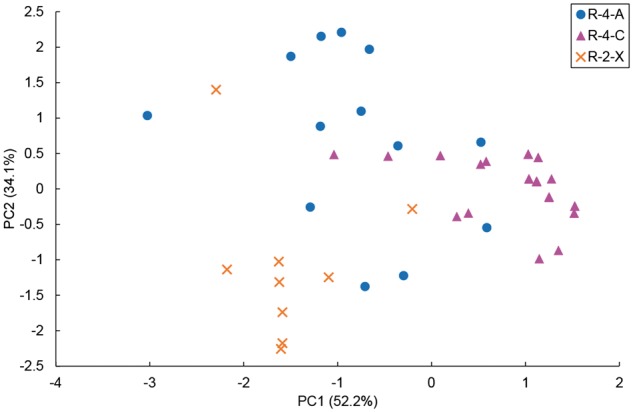
The results of PCA on three HMM scores using Area 1 + 3 + 4 corresponding to three reaction types: R-4-A, R-4-C and R-2-X. The first and second principal components (PC1 and PC2) are plotted and correspond to 52.2% and 34.1% of the variance, respectively

Next, we added nine R-2-X sequences to the dataset to assess the prediction by the pHMMs. The LOOCV accuracy of the prediction using raw HMM scores for the three reaction types was not as high (43/49; 87.8% at maximum; see Supplementary Table S4 for details). The RRSV accuracies were around 80% for most Area selections and many of them were comparable to LOOCV accuracies (Supplementary Table S5). To examine the potential of the pHMMs as predictors, we performed PCA on the three HMM scores. The results for the pHMMs using Area 1 + 3 + 4 showed that the first two principal components corresponded to 86% of the total variance (Fig. 3). As shown in the scatter plot, the three reaction types were somewhat mixed in both components, and the same tendency was observed for pHMMs constructed from other combinations of Areas (Supplementary Fig. S2). Thus, we concluded that it is hard to discriminate the three reaction types with high accuracy using only pHMMs.

### 3.3 Correlation scores

**Fig. 4:**
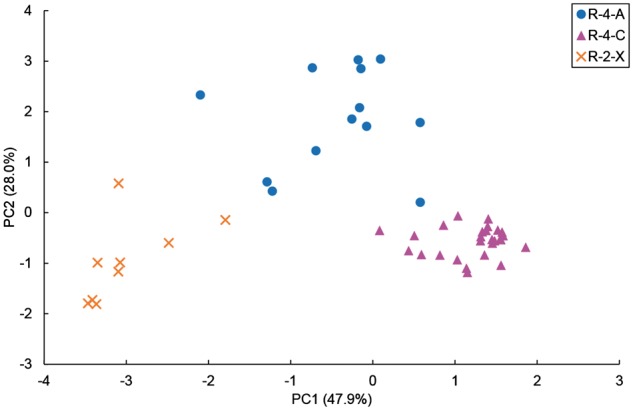
The results of PCA on six scores: three HMM scores using Area 1 + 3 + 4 and three correlation scores for the reaction type-models, R-4-A, R-4-C and R-2-X. The first and second principal components (PC1 and PC2) are plotted and correspond to 47.9% and 28.0% of the variance, respectively

Using the Z-scores (> 3.0) of *MIp* values for each of the three reaction types, R-4-A, R-4-C and R-2-X, as a threshold, 1,075, 1,161 and 1,048 highly correlated residue pairs were identified, respectively. Correlation models of each reaction type were constructed using the frequencies of the residues and residue pairs of the correlated position pairs. Then, correlation scores were calculated as described in 2.4 and we performed PCA on the six scores (three HMM scores from the Areas and three correlation scores) against the models of the three reaction types. The results obtained from the HMM using Area 1 + 3 + 4 showed that the first two principal components corresponded to 76% of the total variance (Fig. 4). As shown in the scatter plot, the three reaction types were almost easy to separate using linear discrimination. In the following analyses, we used pHMMs constructed from Area 1 + 3 + 4 because of its clear result in PCA compared with other Areas (Supplementary Fig. S3).

### 3.4 Classifiers of reaction types

We performed LDA on the six scores, i.e., six dimension input, consisting of the HMM and correlation scores of the R-4-A, R-4-C and R-2-X types, to find the appropriate weight of each score to discriminate the three types and other types (i.e., Rn-2-n/Rn-4-Cn, R-4-C R-2-X bifunctional, R-*-L and S-*-*) of known plant type III PKSs. As a result, three binary classifiers for R-4-A, R-4-C and R-2-X represented by linear combinations of the six scores were obtained. These classifiers were designed to predict whether a query sequence corresponded to that reaction type. We tested the validity of the classifiers against 70 known type III PKSs used as training sets and the results are summarized in Table 1. R-4-A, R-4-C, R-2-X and Rn-2-n/Rn-4-Cn type sequences were all correctly classified as R-4-A/other/other, other/R-4-C/other, other/other/R-2-X and other/other/other by the R-4-A/R-4-C/R-2-X classifiers, respectively. Two bifunctional ‘chalcone synthase and benzalacetone synthase’ enzymes that catalyze both R-4-C and R-2-X types were classified as R-4-C and not as either R-2-X or other. One R-3m-L, two R-4-L and one S-3-L (*Conium maculatum* CPKS5) type PKSs were incorrectly classified as either R-4-A or R-4-C. Sequences of PKSs that mainly use short chain starters (S-*-*) excluding CPKS5 were correctly classified as other types by all the classifiers. In total, the reaction types of 64 of 70 PKSs were correctly classified by the classifiers. 12 type III PKSs that use mainly long chain starters (L-*-A and Lh-4-L) were not used in LDA, but were all correctly classified as others.

**Table 1:** Classification results of LDA using three classifiers

Query	Classifier (R-4-A)		Classifier (R-4-C)		Classifier (R-2-X)		pPAP
	Yes	No	Yes	No	Yes	No	True
R-4-A	13	0	0	13	0	13	13
R-4-C	0	27	27	0	0	27	27
R-2-X	0	9	0	9	9	0	9
Rn-2-n/Rn-4-Cn	0	4	0	4	0	4	4
R-4-C, R-2-X	0	2	2	0	0	2	0
R-3m-L	0	1	1	0	0	1	0
R-4-L	1	1	1	1	0	2	0
S-*-*	0	12	1	11	0	12	11
L-4-A/L-5-A	0	6	0	6	0	6	6
Lh-4-L	0	6	0	6	0	6	6

*Note*: The numbers of correct prediction by pPAP are also shown in the rightmost column.

### 3.5 Prediction of reaction types for plant PKS candidates

**Fig. 5:**
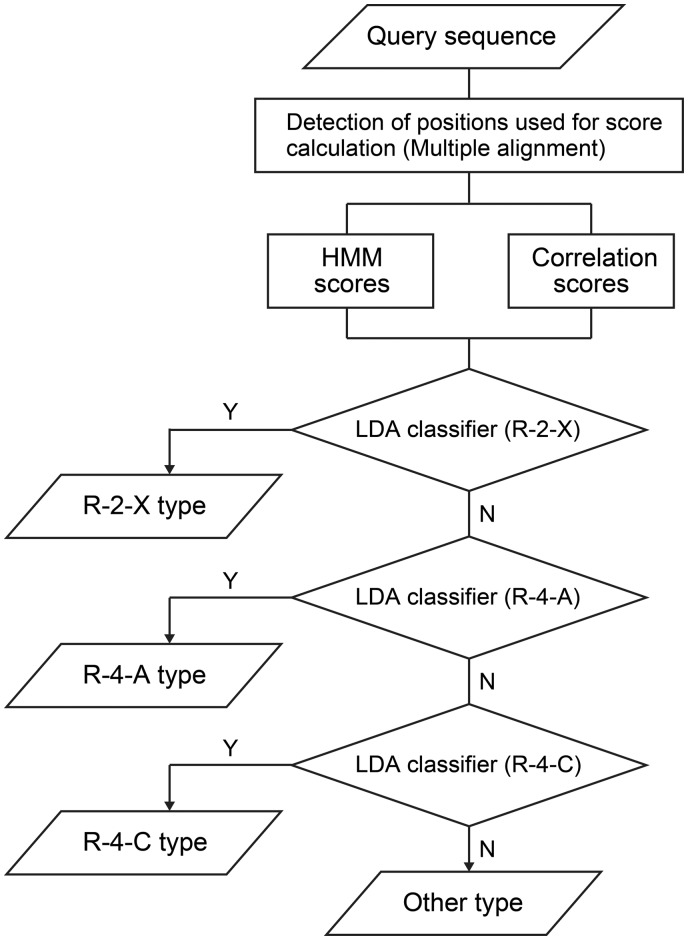
Decision rules for reaction type prediction of plant type III PKSs

Finally, we developed a system predicting four reaction types, R-4-A, R-4-C, R-2-X and other, by successively applying the three classifiers (Fig. 5). The system was named as pPAP (for plant PKS Analysis and Prediction), and is available at ftp://ftp.genome.jp/pub/tools/ppap/. Performance of the prediction system using the above 82 known type III PKSs is shown in Table 1.

We applied pPAP to the prediction of reaction types of 636 plant type III PKS candidate sequences extracted from the KEGG GENES database (Kanehisa *et al.*, 2016) (Supplementary Data). As a result, 105, 190, 8 and 333 sequences were predicted their main reaction types as R-4-A, R-4-C, R-2-X and other, respectively. This result showed fewer R-2-X type-specific sequences in plant genomes compared with R-4-A and R-4-C types (Supplementary Fig. S4), which may be explained by the limited number of families possessing R-2-X type PKSs as shown in Supplementary Figure S1 as well as the availability of only a few genomes from those families. The prediction result included close paralogs with different reaction types, suggesting recent functional alterations of paralogs in different lineages, such as *Medicago truncatula* MTR_3g086260 for other type (Supplementary Fig. S4).

## 4 Discussion

In this study, we have developed a system to predict functions of plant type III PKS sequences. The prediction system is based on a series of LDA on the three HMM and three correlation scores. All R-4-A, R-4-C, R-2-X, Rn-2-n/Rn-4-Cn and L-*-* types, and most of the S-*-* types, were correctly classified into four types by the three classifiers. The system could distinguish close paralogs of different reaction types, such as R-4-C and other types. It could also detect distant paralogs of the R-4-A type. It should be noted that such a function prediction system for paralogous proteins could not be established without prior functional classifications, such as the one used in this study for type III PKS reaction types (Shimizu *et al.*, 2017). We conclude that this system can predict functions for plant type III PKS paralogous genes and that a similar approach could be useful for other paralogous genes that have undergone family-specific duplication events followed by independent acquisition of similar functions (Pichersky and Lewinsohn, 2011).

We used the Areas corresponding to structural differences between R-4-A and R-4-C types for constructing pHMMs. LOOCV and RRSV showed that HMMs from Area 1 + 3 + 4 were the best to discriminate R-4-A and R-4-C types, while utilization of Area 2 did not contribute to a better discrimination. This was because some R-4-A type PKSs, such as *Vitis vinifera*, *Arachis hypogaea* and *Sorghum bicolor* STSs, share the motif ‘TTSGVDM’ in Area 2 with R-4-C type PKSs (Fig. 2). These STSs do not have any important residue changes in Area 2 that lead to a structural alteration and to a switch from Claisen (R-4-C type) to aldol (R-4-A type) cyclizations called the ‘aldol switch’ (Austin *et al.*, 2004). The 3D crystal structure of *A*. *hypogaea* STS, instead, suggested that another residue, Met98, in Area 1 likely contributes to the structural difference in Area 2, which affects the hydrogen-bond network required in the ‘aldol switch’ (Shomura *et al.*, 2005). Hence, the structural changes in R-4-A types from R-4-C types do not necessarily require residue changes in Area 2.

A detailed analysis of Area 2 also indicated a possible reason why correlation scores are necessary in addition to the HMM scores. For example, in Area 2 of *Polygonum cuspidatum* STS, *Rheum tataricum* STS and *Malus *×* domestica* BIS, Thr132 is replaced by Ile or Ala, which may lead to the residue changes in other positions to develop a different aldol cyclization system to the ‘aldol switch’ type hydrogen-bond-network (Cook *et al.*, 2010). These variations in the structural environments in R-4-A type explain the need for the correlation score, while pHMMs showed the power to discriminate R-4-C types due to the high conservation of R-4-C types in the Areas.

Another example is *H*. *macrophylla* STCS (R-4-A) whose prediction using only the HMM scores failed. It was initially considered to catalyze the *in vivo* and *in vitro* production of stilbenecarboxylic acids such as lunularic acid, hydrangeic acid and 5-hydroxylunularic acid via intramolecular ‘nondecarboxylative’ aldol cyclization of tetraketide intermediates, which differs from the ‘decarboxylative’ aldol cyclization commonly used by other R-4-A type PKSs such as STS (Eckermann *et al.*, 2003). A hypothesis was proposed that the aldol cyclization required for the production of stilbenecarboxylic acids occurs spontaneously from linear intermediates reopened from tetraketide lactone products that result from R-4-L type reactions (Austin *et al.*, 2004). This may explain that the Areas of STCS do not have the same sequence features as other R-4-A type PKSs and cannot be used for its reaction type prediction using only HMM scores.

Despite the high accuracy achieved by our prediction system, the predictions were inherently difficult in CTASs (R-4-L). *H*. *macrophylla* var. *thunbergii* CTAS was classified as an R-4-A, which was most likely because of the extremely high similarity (only a five residue difference) between the STCS and CTAS. It is also possible that the lactone products of the CTAS are spontaneously converted to stilbenecarboxylic acids in a solution state with a long reaction time (Austin *et al.*, 2004), which may account for the CTAS being classified as an R-4-A. *Rubus idaeus* CTAS was incorrectly classified as an R-4-C type. This classification was also most likely due to the extremely high similarity (only a four residue difference) between *R*. *idaeus* CTAS and *R*. *idaeus* PKS1 (CHS) (Zheng *et al.*, 2001). Intramolecular lactonizations (e.g. R-3-L, R-4-L and S-3-L) occur as derailment reactions of R-4-A type or R-4-C type enzymes (Abe and Morita, 2010) and, therefore, a few point mutations affecting the main reactions may yield lactone-specific enzymes, which are hard to discriminate by our approach and would require more specific classifiers. R-4-C and R-2-X bifunctional type sequences were hit only by the R-4-C classifier, probably due to the acquisition of their R-2-X functions as a result of multifunctionalization from R-4-C and the main function of these sequences are still R-4-C. In such a case, our predictor assigns a possible main reaction type to the multifunctional enzyme. Overall, we obtained good classifiers to discriminate the three types and others.

## Supplementary Material

Supplementary Data
