# Supplementary material for: Discriminating the reaction types of plant type III polyketide synthases
Source: Bioinformatics. 2017 Mar 6;33(13):1937–43. doi: 10.1093/bioinformatics/btx112 (PMC5870536; doi:10.1093/bioinformatics/btx112)
Supplement: Supplementary Data [file btx112_supp.zip › Supplementary Data.docx]

Table S1. List of known plant type III PKSs and their reaction types. STS: Stilbene synthase, BIS: Biphenyl synthase, BBS: Bibenzyl synthase, STCS: Stilbenecarboxylate synthase, CHS: Chalcone synthase, BPS: Benzophenone synthase, HEDS: Homoeriodictyol/eriodictyol synthase, BAS: Benzalacetone synthase, DCS: Diketide-CoA synthase, CURS: Curcumin synthase, CUS: Curcuminoid synthase, ACS: Acridone synthase, QNS: Quinolone synthase, CTAS: Coumaroyltriacetic acid synthase, VPS: Phlorisovalerophenone synthase, OLS: Olivetol synthase, 2PS: 2-Pyrone synthase, PCS: Pentaketide chromone synthase, HKS: Hexaketide synthase, ALS: Aloesone synthase, OKS: Octaketide synthase, ARS: Alkylresorcinol synthase, ARAS: Alkylresorcylic acid synthase, ORS: 2′-oxoalkylresorcinol synthase, ASCL: Anther-specific chalcone synthase-like enzyme.

| Reaction type | PKS name | GenBank Accession |
| --- | --- | --- |
| R-4-A | *Vitis vinifera* STS2 | CAA54221 |
| R-4-A | *Arachis hypogaea* STS | BAA78617 |
| R-4-A | *Rheum tataricum* STS | AAP13782 |
| R-4-A | *Polygonum cuspidatum* PcPKS5 (STS) | AEE25896 |
| R-4-A | *Sorghum bicolor* STS1 | AAL49965 |
| R-4-A | *Pinus strobus* STS2 | CAA87013 |
| R-4-A | *Pinus sylvestris* STS | CAA43165 |
| R-4-A | *Psilotum nudum* PnI (STS) | BAA87925 |
| R-4-A | *Psilotum nudum* PnL (STS) | BAA87924 |
| R-4-A | *Malus* x *domestica* BIS4 | AEX88418 |
| R-4-A | *Bromheadia finlaysoniana* BBS | CAA10514 |
| R-4-A | *Phalaenopsis* sp. BBS | CAA56276 |
| R-4-A | *Hydrangea macrophylla* STCS1 | AAN76182 |
| R-4-C | *Vitis vinifera* CHS | CAA53583 |
| R-4-C | *Garcinia mangostana* CHS | ACM62742 |
| R-4-C | *Arachis hypogaea* CHS1 | AAO32821 |
| R-4-C | *Medicago sativa* CHS2 | AAA02824 |
| R-4-C | *Humulus lupulus* CHS | CAC19808 |
| R-4-C | *Rubus idaeus* PKS1 (CHS) | AAK15174 |
| R-4-C | *Citrus sinensis* CHS1 | BAA81663 |
| R-4-C | *Ruta graveolens* CHS1 | CAC14059 |
| R-4-C | *Arabidopsis thaliana* CHS | AAA32771 |
| R-4-C | *Rheum palmatum* CHS1 | ABB13607 |
| R-4-C | *Polygonum cuspidatum* PKS3 (CHS) | AEE25895 |
| R-4-C | *Hydrangea macrophylla* var. *thunbergii* CHS | BAA32732 |
| R-4-C | *Scutellaria baicalensis* CHS | BAA23373 |
| R-4-C | *Solanum lycopersicum* CHS1 | CAA38980 |
| R-4-C | *Conium maculatum* CPKS2 (CHS) | AKR53955 |
| R-4-C | *Petroselinum crispum* CHS | CAA24779 |
| R-4-C | *Gerbera hybrida* CHS1 | CAA86218 |
| R-4-C | *Gerbera hybrida* CHS3 | CAA86220 |
| R-4-C | *Bromheadia finlaysoniana* CHS3 | AAB62874 |
| R-4-C | *Sorghum bicolor* CHS2 | AAD41874 |
| R-4-C | *Pinus densiflora* PdCHSX | BAA94594 |
| R-4-C | *Psilotum nudum* PnJ (CHS) | BAA87922 |
| R-4-C | *Physcomitrella patens* CHS | ABB84527 |
| R-4-C | *Plagiochasma appendiculatum* CHS | AHY39238 |
| R-4-C | *Garcinia mangostana* BPS | AEI27291 |
| R-4-C | *Hypericum androsaemum* BPS | AAL79808 |
| R-4-C | *Hordeum vulgare* HEDS | CAA70435 |
| R-2-X | *Polygonum cuspidatum* PcPKS2 (BAS) | ABY47641 |
| R-2-X | *Rheum palmatum* BAS | AAK82824 |
| R-2-X | *Curcuma longa* DCS | BAH56225 |
| R-2-X | *Wachendorfia thyrsiflora* WtPKS1 | AAW50921 |
| R-2d-X | *Curcuma longa* CURS1 | BAH56226 |
| R-2d-X | *Curcuma longa* CURS2 | BAH85780 |
| R-2d-X | *Curcuma longa* CURS3 | BAH85781 |
| R-2d-X | *Wachendorfia thyrsiflora* WtPKS2 | AAW50922 |
| R-2-X, R-2d-X | *Oryza sativa* CUS | BAC79571 |
| R-4-C, R-2-X | *Polygonum cuspidatum* PcPKS1 (CHS/BAS) | ABK92281 |
| R-4-C, R-2-X | *Rubus idaeus* RiPKS4 (CHS/BAS) | ABV54602 |
| Rn-4-Cn | *Ruta graveolens* ACS1 (ACS3) | CAC14056 |
| Rn-4-Cn, Rn-2-n | *Citrus microcarpa* ACS | BAO05327 |
| Rn-2-n | *Citrus microcarpa* QNS | BAO05328 |
| Rn-2-n, Rn-4-Cn | *Huperzia serrata* HsPKS1 | ABI94386 |
| R-4-L | *Rubus idaeus* RiPKS3 (CTAS) | AAK15176 |
| R-4-L | *Hydrangea macrophylla* var. *thunbergii* CTAS | BAA32733 |
| R-3m-L | *Pinus strobus* PstrCHS2 | CAA05214 |
| Sb-4-C | *Humulus lupulus* VPS | BAA29039 |
| Sb-4-C | *Psilotum nudum* PnP (VPS) | BAA87923 |
| S-4-A | *Cannabis sativa* OLS | BAG14339 |
| Sb-3-L | *Gerbera hybrida* 2PS | CAA86219 |
| S-3-L | *Conium maculatum* CPKS5 | AKR53956 |
| Sc-5-C+ | *Aloe arborescens* PCS | AAX35541 |
| S-6-AL | *Plumbago indica* HKS | BAF44539 |
| S-6-AL | *Drosophyllum lusitanicum* HKS | ABQ59603 |
| S-7-A+ | *Rheum palmatum* ALS | AAS87170 |
| Sc-7-A+ | *Aloe arborescens* AaPKS3 | ABS72373 |
| Sc-8-AL+ | *Aloe arborescens* PKS5 (OKS) | ACR19998 |
| S-8-AL+ | *Hypericum perforatum* HpPKS2 (OKS) | ACF37207 |
| L-4-A | *Brachypodium distachyon* BdARS | KQJ89844 |
| L-4-A | *Oryza sativa* Os05g12180 (ARS) | AAT44238 |
| L-4-A | *Oryza sativa* Os10g07040 (ARAS/ARS) | AAK95679 |
| L-4-A | *Sorghum bicolor* ARS2 | XP_002441839 |
| L-4-A | *Oryza sativa* Os10g08620 (ARAS/ARS) | AAN04188 |
| L-5-A | *Physcomitrella patens* PpORS | ABU87504 |
| Lh-4-L | *Oryza sativa* OsPKS1 (ASCL) | AAP54339 |
| Lh-4-L | *Hypericum perforatum* HpPKS1 (ASCL) | AFN26935 |
| Lh-4-L | *Arabidopsis thaliana* PKS-B (ASCL) | NP_567971 |
| Lh-4-L | *Nicotiana tabacum* NtPKS1 (ASCL) | CAA74846 |
| Lh-4-L | *Arabidopsis thaliana* PKS-A (ASCL) | NP_171707 |
| Lh-4-L | *Physcomitrella patens* PpASCL | XP_001781520 |

Table S2. LOOCV accuracy of discriminating two reaction types, R-4-A and R-4-C, by the two pHMMs corresponding to selected combinations of areas.

| Areas used for  pHMM construction | Reaction type of test set | | |
| --- | --- | --- | --- |
|  | R-4-A | R-4-C | Total |
| 1 | 6/13 (46.2%) | 25/27 (92.6%) | 31/40 (77.5%) |
| 2 | 9/13 (69.2%) | 27/27 (100.0%) | 36/40 (90.0%) |
| 3 | 9/13 (69.2%) | 27/27 (100.0%) | 36/40 (90.0%) |
| 4 | 10/13 (76.9%) | 27/27 (100.0%) | 37/40 (92.5%) |
| 1+2 | 10/13 (76.9%) | 26/27 (96.3%) | 36/40 (90.0%) |
| 1+3 | 11/13 (84.6%) | 25/27 (92.6%) | 36/40 (90.0%) |
| 1+4 | 12/13 (92.3%) | 27/27 (100.0%) | 39/40 (97.5%) |
| 2+3 | 9/13 (69.2%) | 27/27 (100.0%) | 36/40 (90.0%) |
| 2+4 | 11/13 (84.6%) | 27/27 (100.0%) | 38/40 (95.0%) |
| 3+4 | 12/13 (92.3%) | 26/27 (96.3%) | 38/40 (95.0%) |
| 1+2+3 | 9/13 (69.2%) | 27/27 (100.0%) | 36/40 (90.0%) |
| 1+2+4 | 11/13 (84.6%) | 27/27 (100.0%) | 38/40 (95.0%) |
| 1+3+4 | 12/13 (92.3%) | 27/27 (100.0%) | 39/40 (97.5%) |
| 2+3+4 | 9/13 (69.2%) | 27/27 (100.0%) | 36/40 (90.0%) |
| 1+2+3+4 | 9/13 (69.2%) | 27/27 (100.0%) | 36/40 (90.0%) |
| whole sequence | 9/13 (69.2%) | 27/27 (100.0%) | 36/40 (90.0%) |

Table S3. RRSV accuracy of discriminating two reaction types, R-4-A and R-4-C, by the two pHMMs corresponding to selected combinations of areas.

| Areas used for  pHMM construction | Reaction type of test-set | | |
| --- | --- | --- | --- |
|  | R-4-A | R-4-C | Total |
| 1 | 41.7% | 87.7% | 72.7% |
| 2 | 58.3% | 98.5% | 85.4% |
| 3 | 58.3% | 93.1% | 81.8% |
| 4 | 75.0% | 93.8% | 87.7% |
| 1+2 | 58.3% | 93.8% | 82.3% |
| 1+3 | 80.0% | 92.3% | 88.3% |
| 1+4 | 85.0% | 96.9% | 93.0% |
| 2+3 | 70.0% | 96.9% | 88.2% |
| 2+4 | 86.7% | 96.9% | 93.6% |
| 3+4 | 90.0% | 96.9% | 94.7% |
| 1+2+3 | 53.3% | 97.7% | 83.3% |
| 1+2+4 | 78.3% | 96.9% | 90.9% |
| 1+3+4 | 90.0% | 96.9% | 94.7% |
| 2+3+4 | 53.3% | 95.4% | 81.7% |
| 1+2+3+4 | 43.3% | 96.2% | 79.0% |
| whole sequence | 46.7% | 96.9% | 80.6% |

Table S4. LOOCV accuracy of discriminating three reaction types, R-4-A, R-4-C, and R-2-X, by the three pHMMs corresponding to selected combinations of areas.

| Areas used for  pHMM construction | Reaction type of test-set | | | |
| --- | --- | --- | --- | --- |
|  | R-4-A | R-4-C | R-2-X | Total |
| 1 | 4/13 (30.8%) | 23/27 (85.2%) | 6/9 (66.7%) | 33/49 (67.3%) |
| 2 | 6/13 (46.2%) | 27/27 (100.0%) | 7/9 (77.8%) | 40/49 (81.6%) |
| 3 | 6/13 (46.2%) | 26/27 (96.3%) | 8/9 (88.9%) | 40/49 (81.6%) |
| 4 | 9/13 (69.2%) | 26/27 (96.3%) | 6/9 (66.7%) | 41/49 (83.7%) |
| 1+2 | 8/13 (61.5%) | 26/27 (96.3%) | 8/9 (88.9%) | 42/49 (85.7%) |
| 1+3 | 8/13 (61.5%) | 25/27 (92.6%) | 8/9 (88.9%) | 41/49 (83.7%) |
| 1+4 | 9/13 (69.2%) | 26/27 (96.3%) | 7/9 (77.8%) | 42/49 (85.7%) |
| 2+3 | 7/13 (53.8%) | 27/27 (100.0%) | 9/9 (100.0%) | 43/49 (87.8%) |
| 2+4 | 9/13 (69.2%) | 27/27 (100.0%) | 7/9 (77.8%) | 43/49 (87.8%) |
| 3+4 | 8/13 (61.5%) | 26/27 (96.3%) | 7/9 (77.8%) | 41/49 (83.7%) |
| 1+2+3 | 7/13 (53.8%) | 27/27 (100.0%) | 8/9 (88.9%) | 42/49 (85.7%) |
| 1+2+4 | 9/13 (69.2%) | 27/27 (100.0%) | 7/9 (77.8%) | 43/49 (87.8%) |
| 1+3+4 | 8/13 (61.5%) | 27/27 (100.0%) | 7/9 (77.8%) | 42/49 (85.7%) |
| 2+3+4 | 7/13 (53.8%) | 27/27 (100.0%) | 8/9 (88.9%) | 42/49 (85.7%) |
| 1+2+3+4 | 7/13 (53.8%) | 27/27 (100.0%) | 8/9 (88.9%) | 42/49 (85.7%) |
| whole sequence | 9/13 (69.2%) | 27/27 (100.0%) | 6/9 (66.7%) | 42/49 (85.7%) |

Table S5. RRSV accuracy of discriminating three reaction types, R-4-A, R-4-C, and R-2-X, by the three pHMMs corresponding to selected combinations of areas.

| Areas used for  pHMM construction | Reaction type of test-set | | | |
| --- | --- | --- | --- | --- |
|  | R-4-A | R-4-C | R-2-X | Total |
| 1 | 26.7% | 81.5% | 67.5% | 64.4% |
| 2 | 45.0% | 98.5% | 75.0% | 80.0% |
| 3 | 31.7% | 92.3% | 70.0% | 72.1% |
| 4 | 65.0% | 96.2% | 65.0% | 82.2% |
| 1+2 | 48.3% | 94.6% | 80.0% | 79.7% |
| 1+3 | 53.3% | 92.3% | 80.0% | 79.7% |
| 1+4 | 70.0% | 96.2% | 72.5% | 84.9% |
| 2+3 | 51.7% | 93.8% | 87.5% | 81.5% |
| 2+4 | 70.0% | 96.9% | 77.5% | 86.2% |
| 3+4 | 61.7% | 96.9% | 70.0% | 82.6% |
| 1+2+3 | 41.7% | 97.7% | 82.5% | 80.0% |
| 1+2+4 | 68.3% | 96.9% | 77.5% | 85.8% |
| 1+3+4 | 60.0% | 95.4% | 72.5% | 81.8% |
| 2+3+4 | 43.3% | 96.9% | 85.0% | 80.5% |
| 1+2+3+4 | 40.0% | 96.9% | 82.5% | 79.2% |
| whole sequence | 40.0% | 96.9% | 65.0% | 76.0% |

**
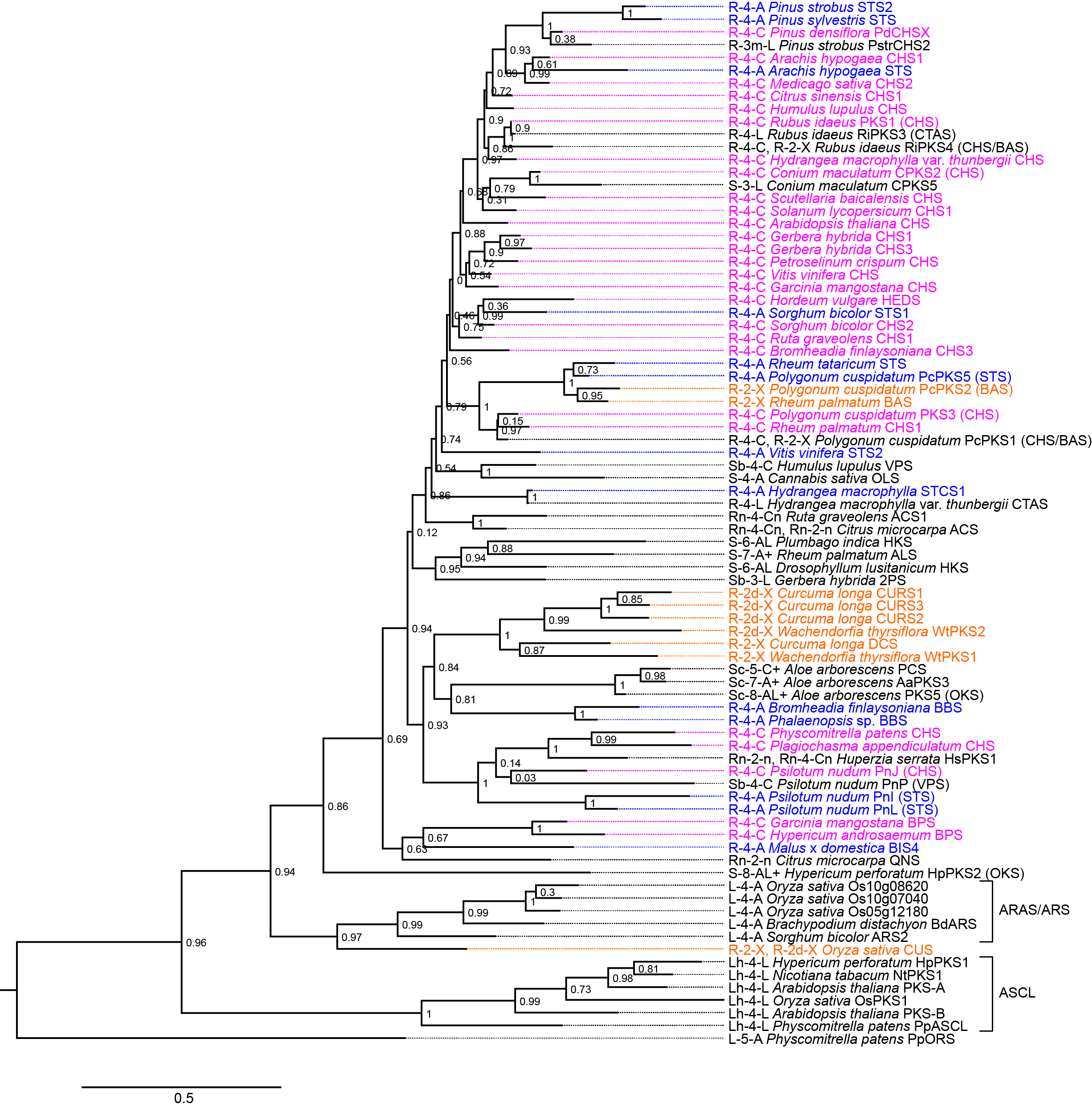
**Fig. S1 Phylogenetic tree of known plant type III PKSs. Representative reaction types, species, and PKS names are indicated at the right. Colors correspond to reaction types: blue, R-4-A; magenta, R-4-C; orange, R-2-X/R-2d-X; black, Others.


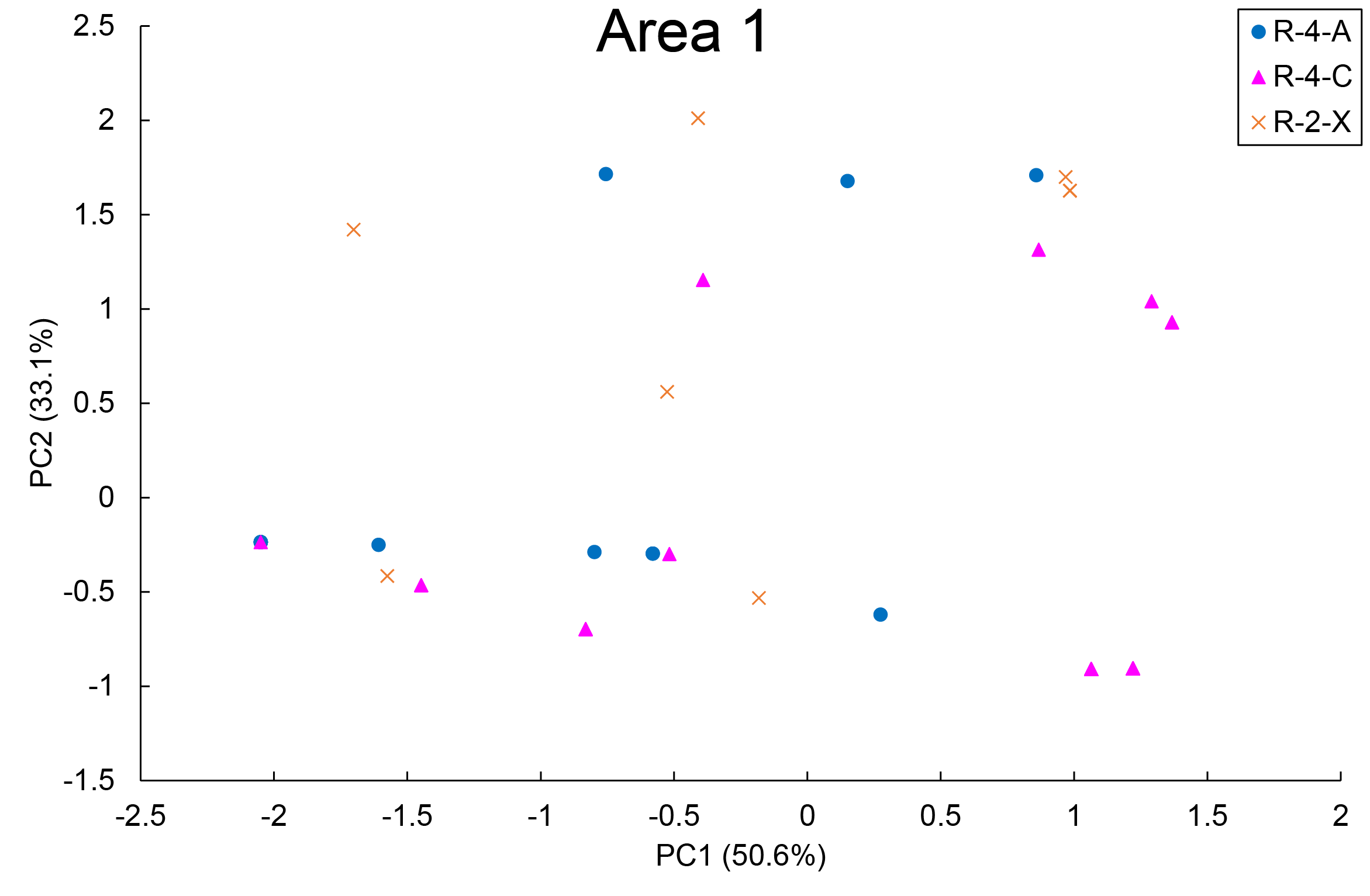

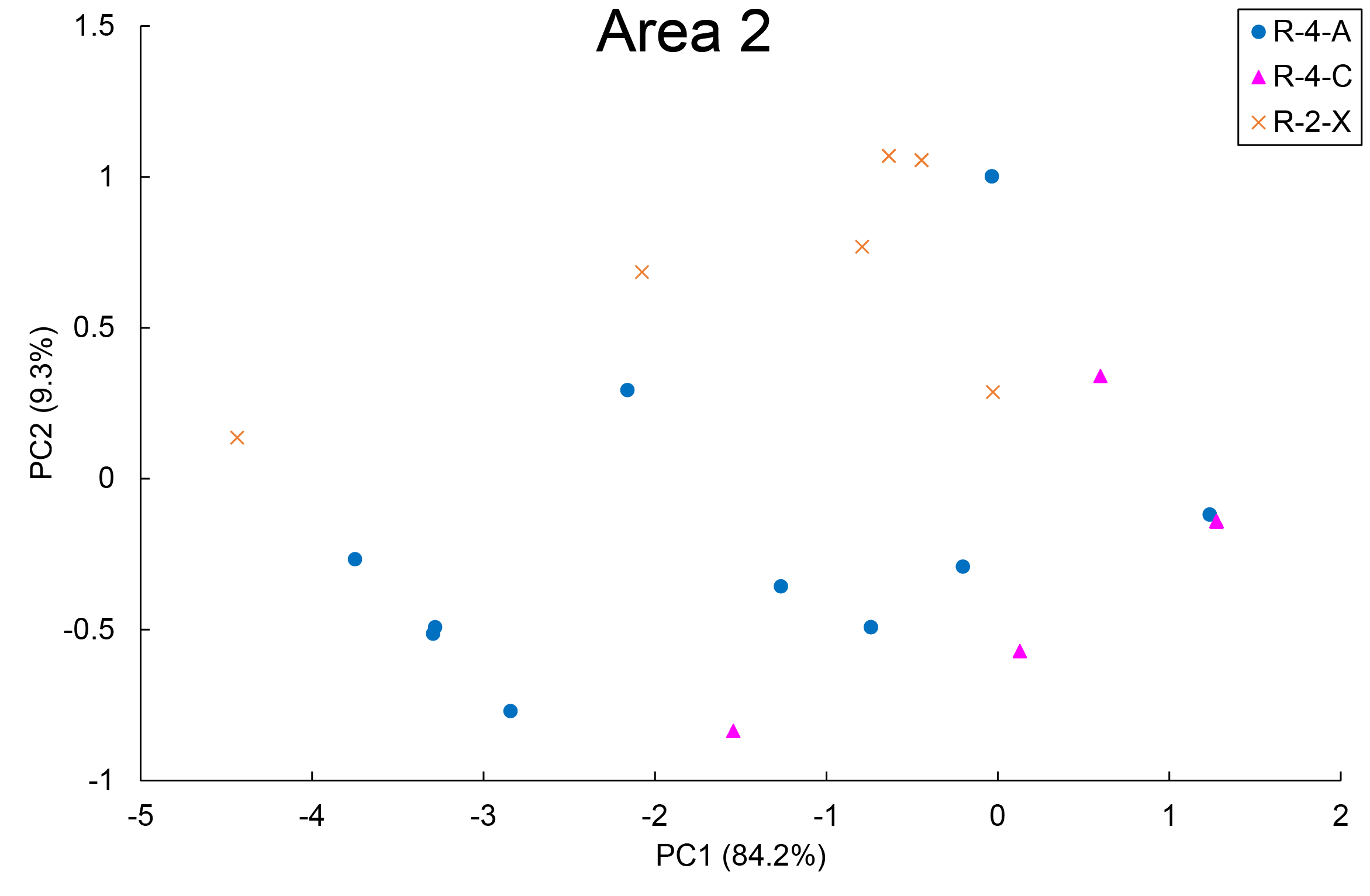


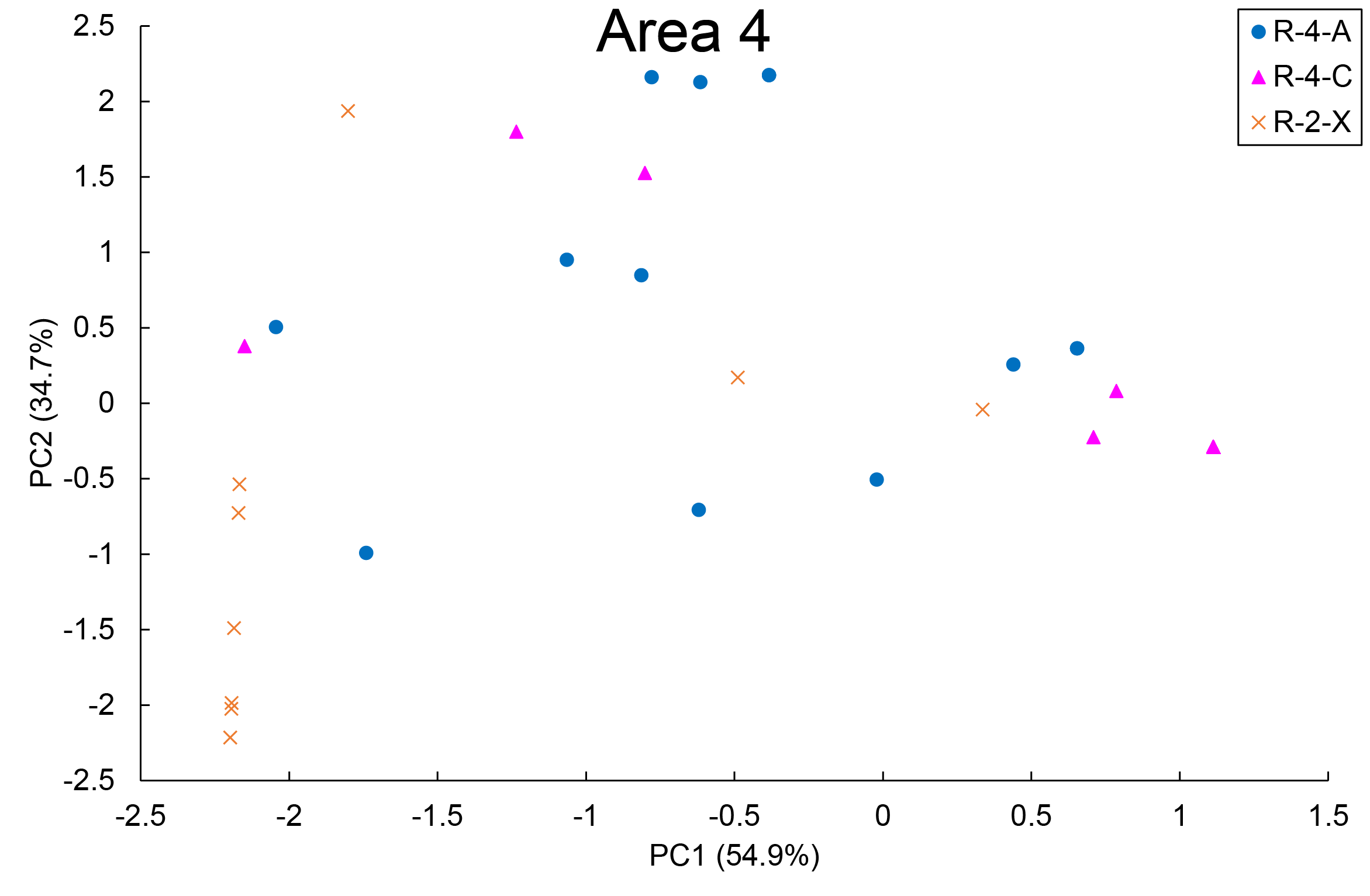

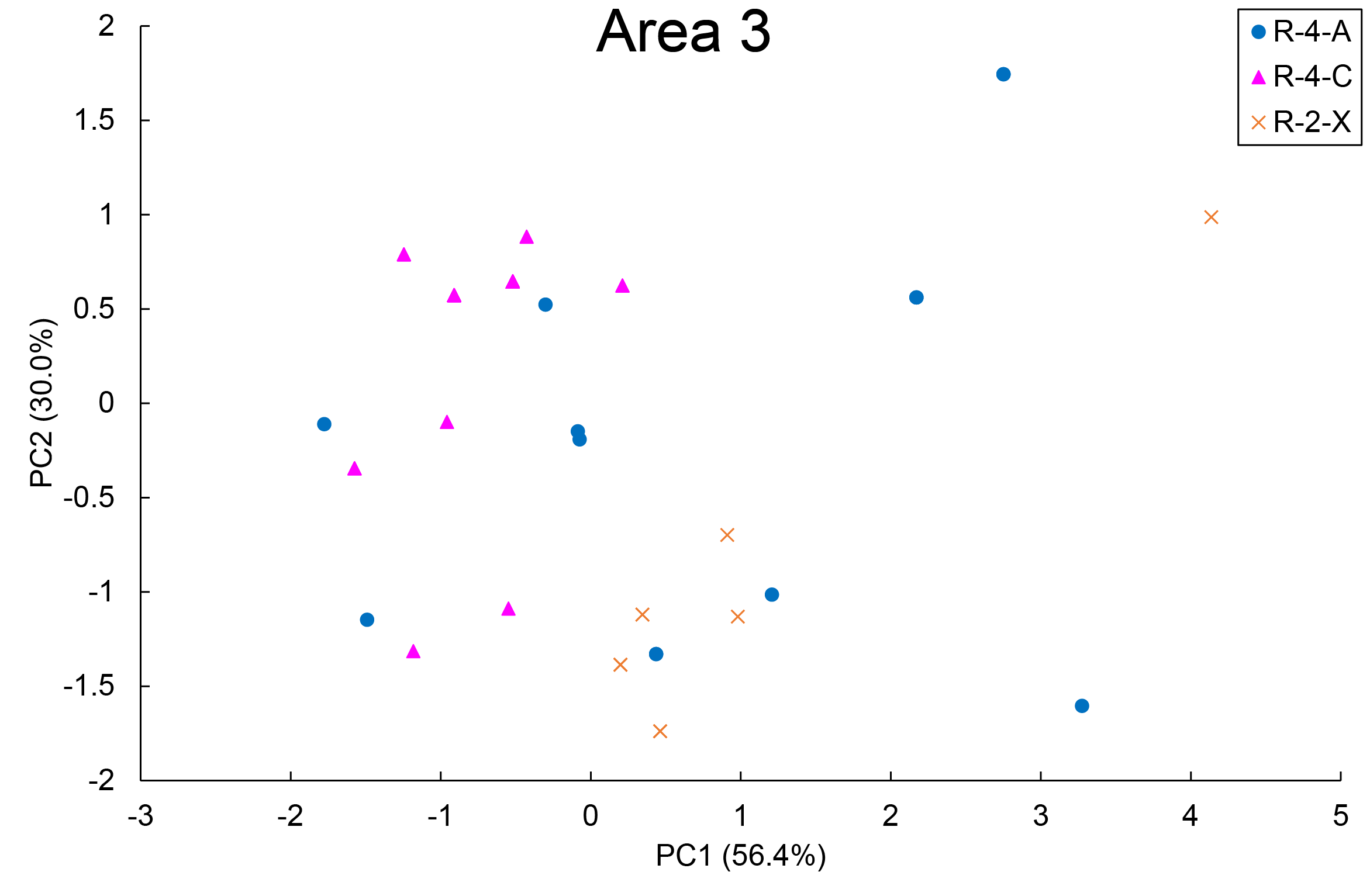


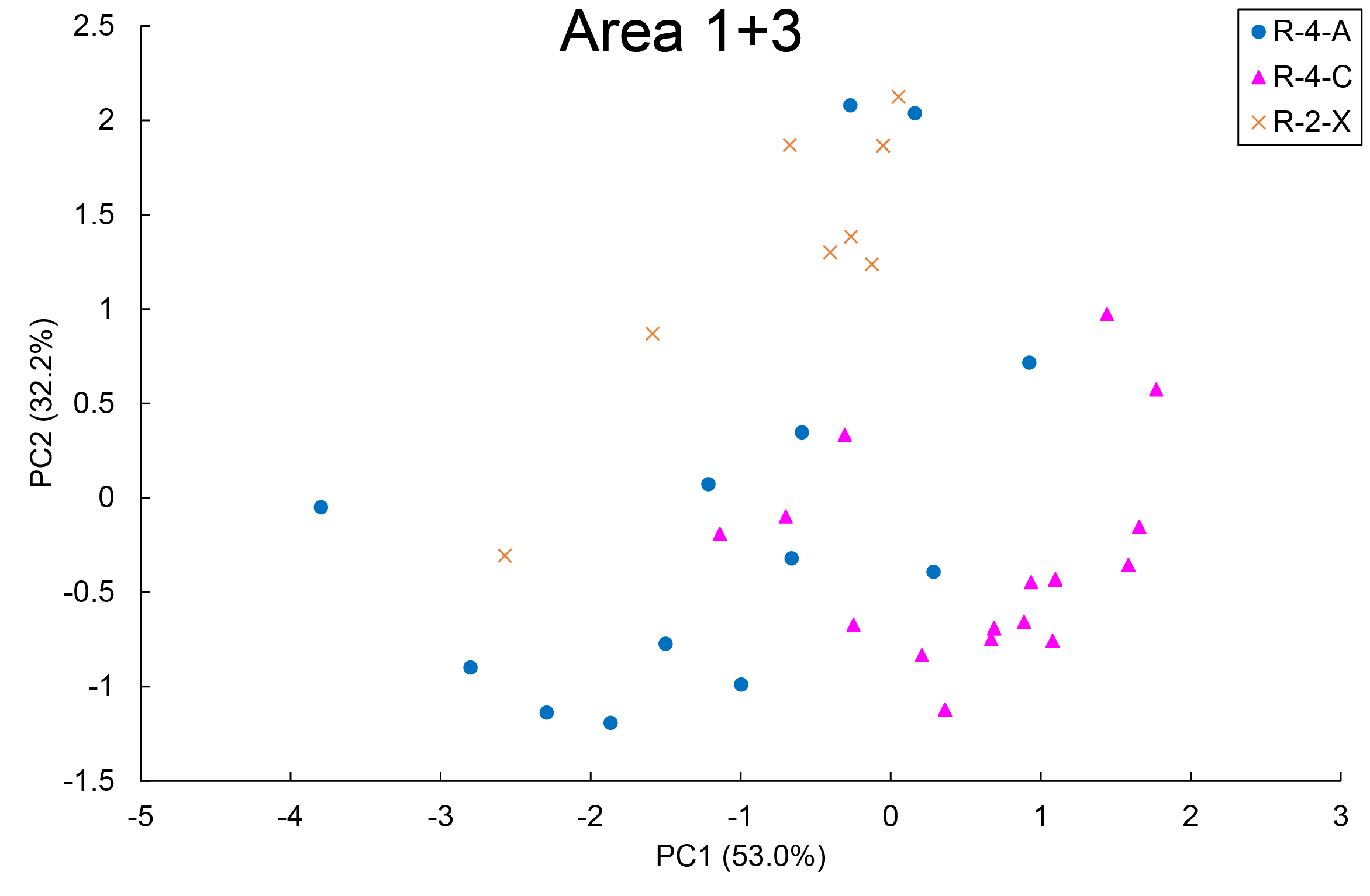

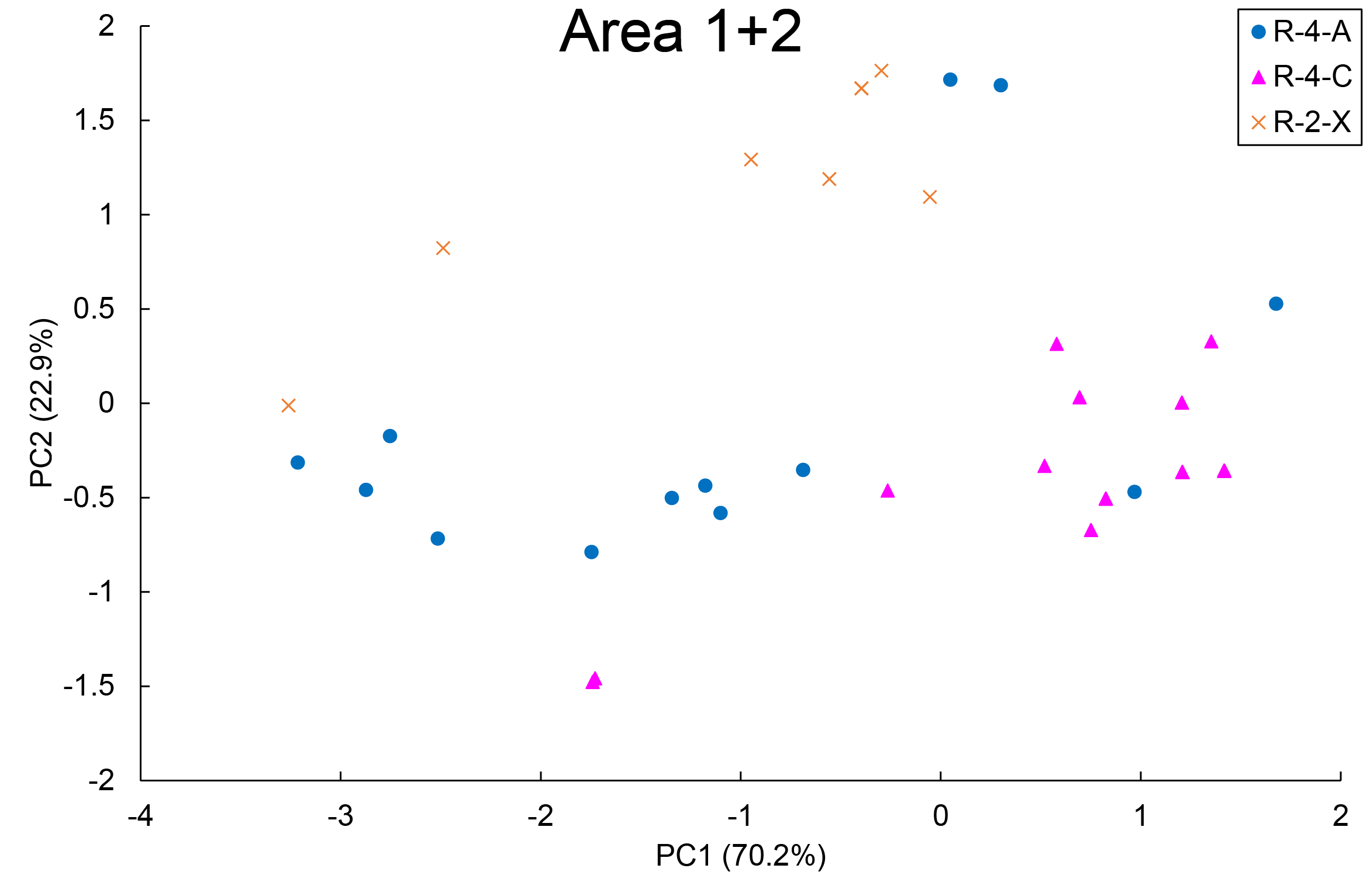


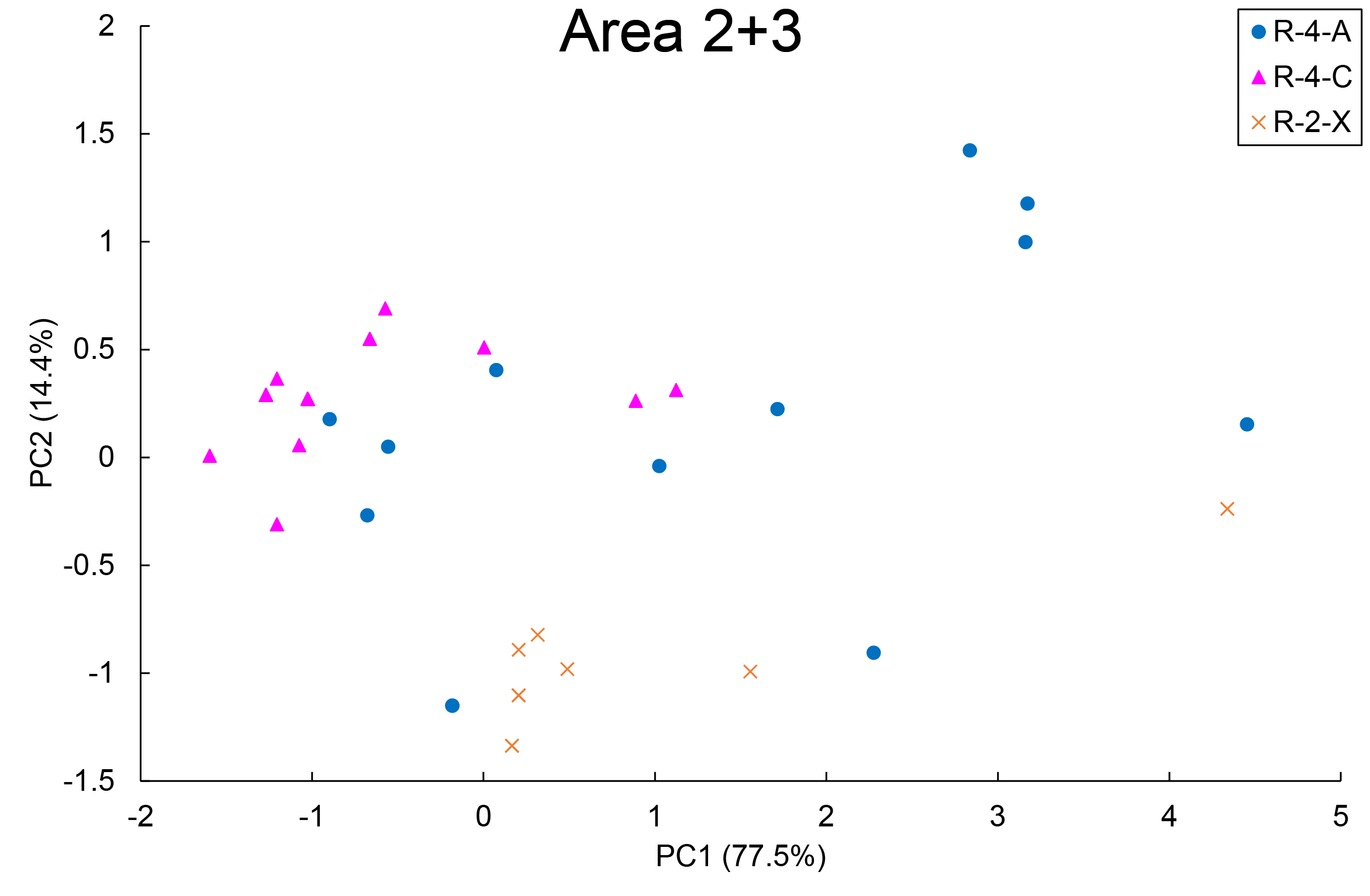

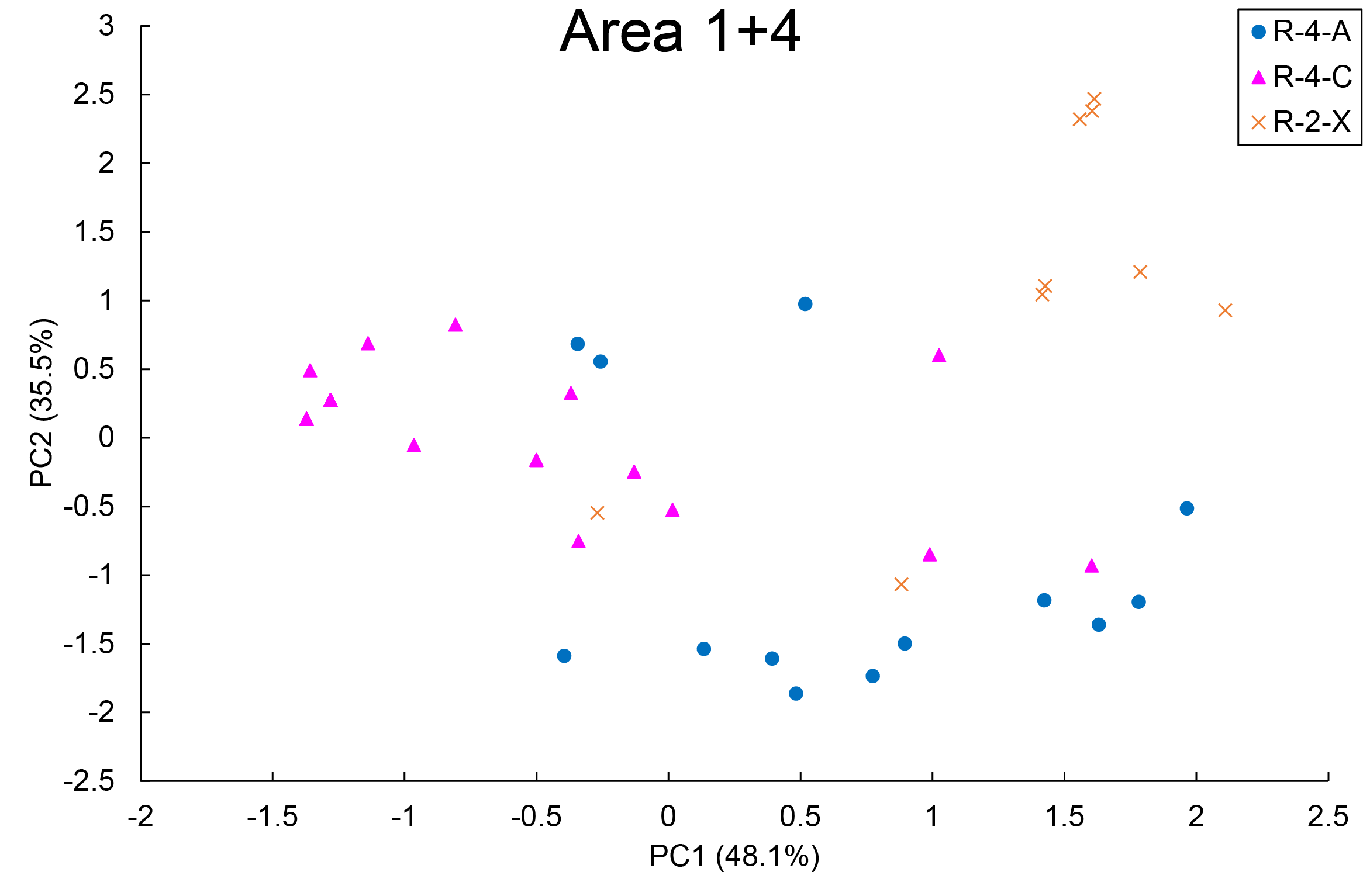


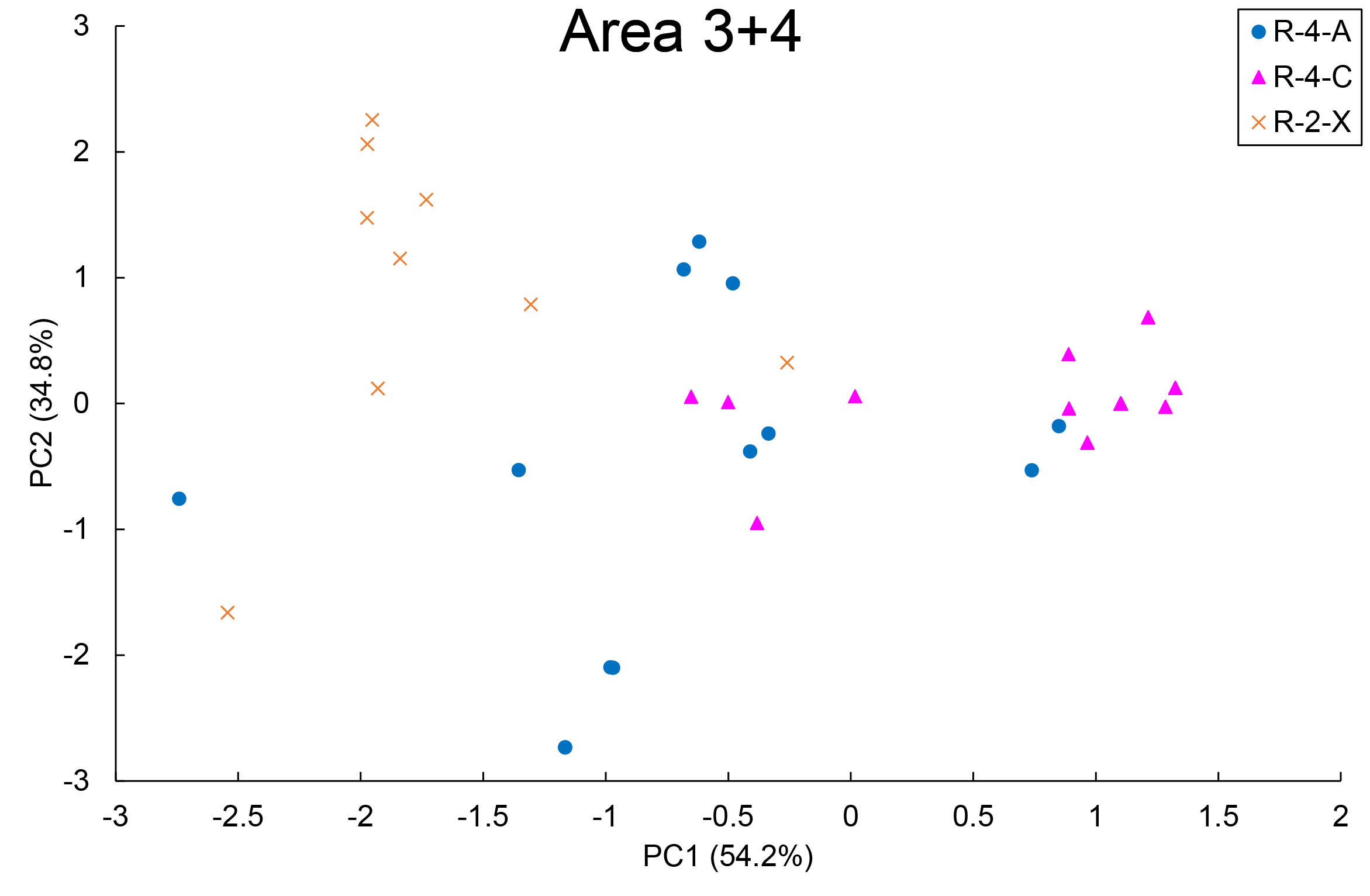

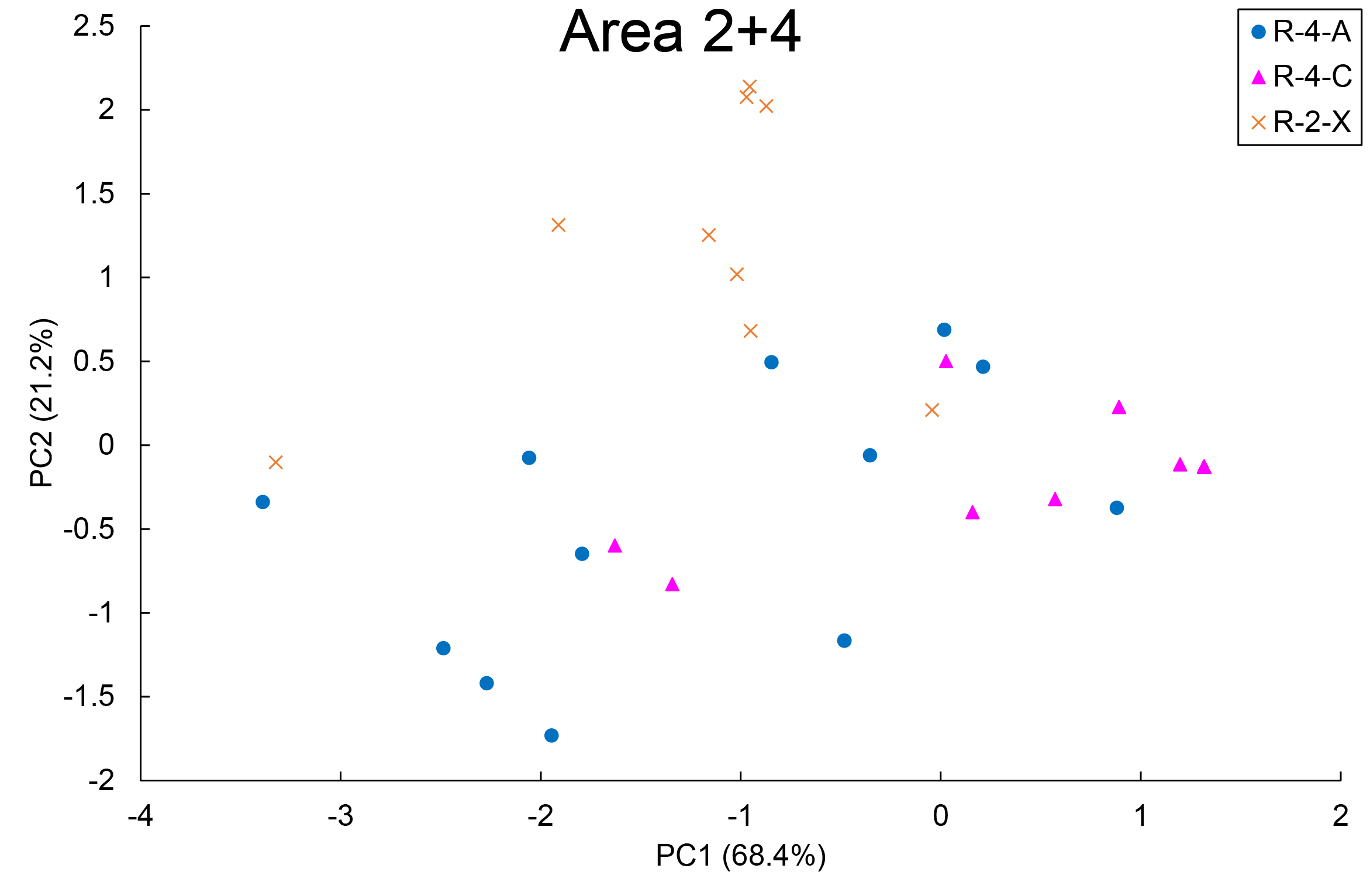


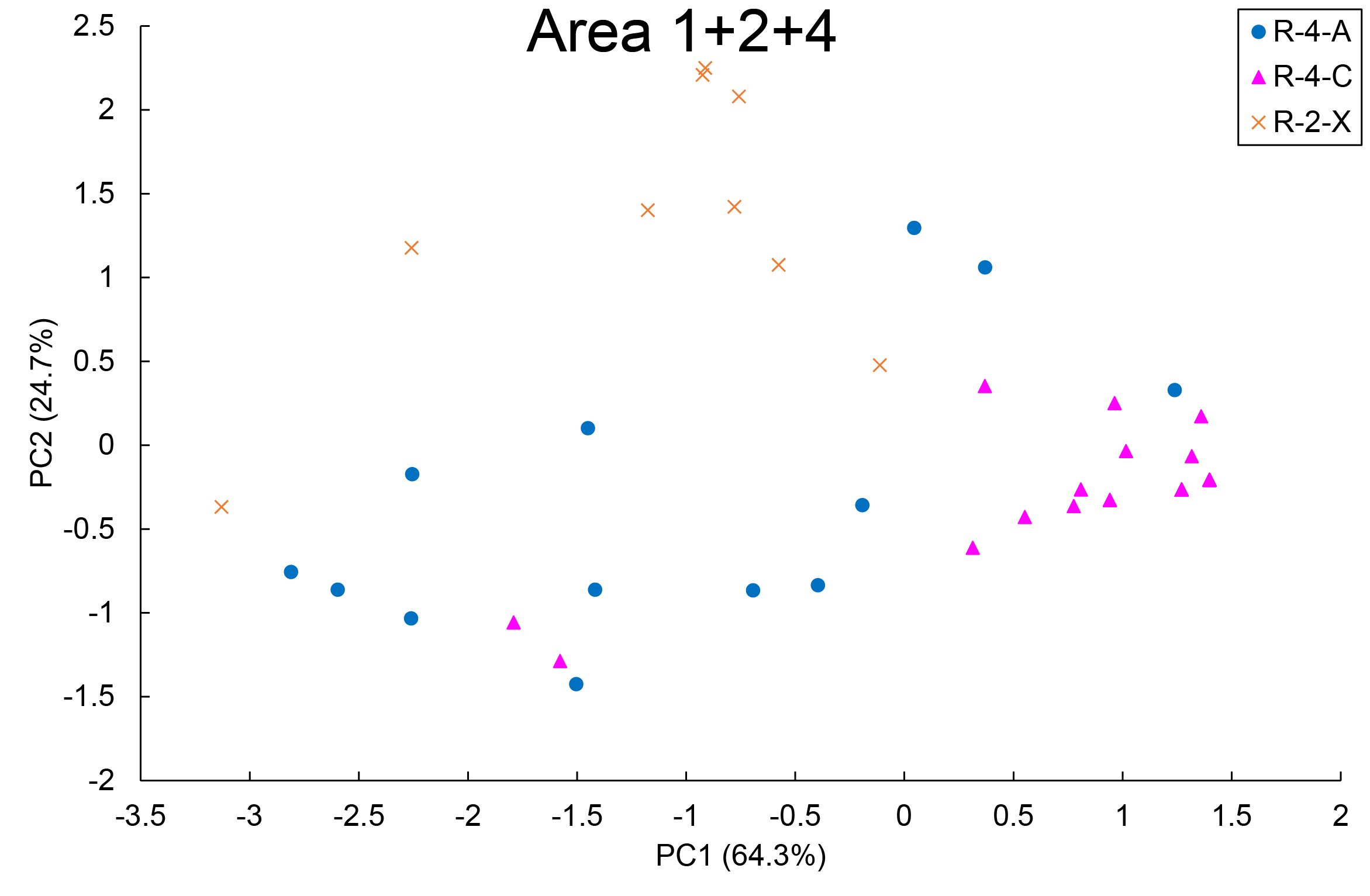

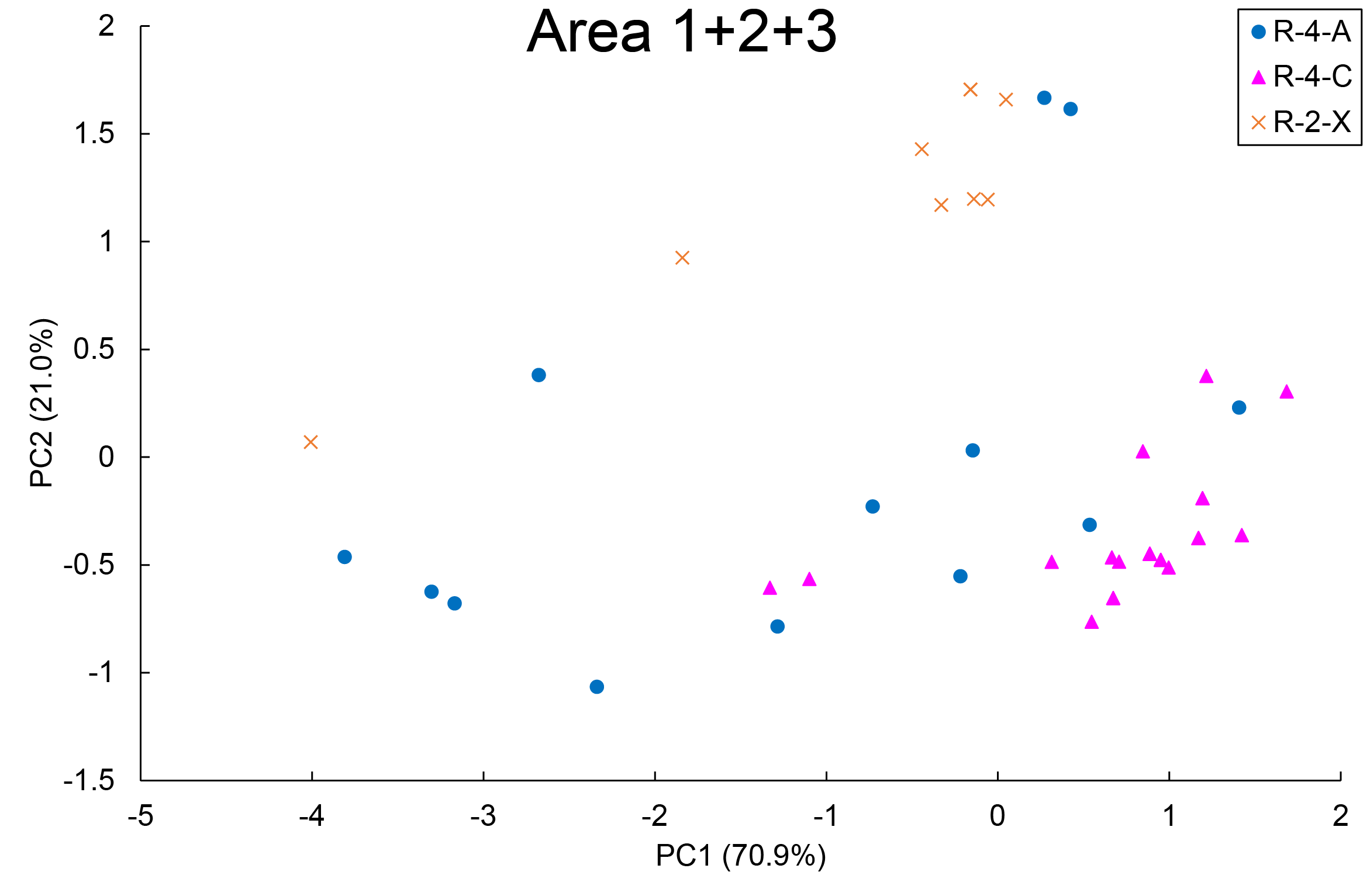


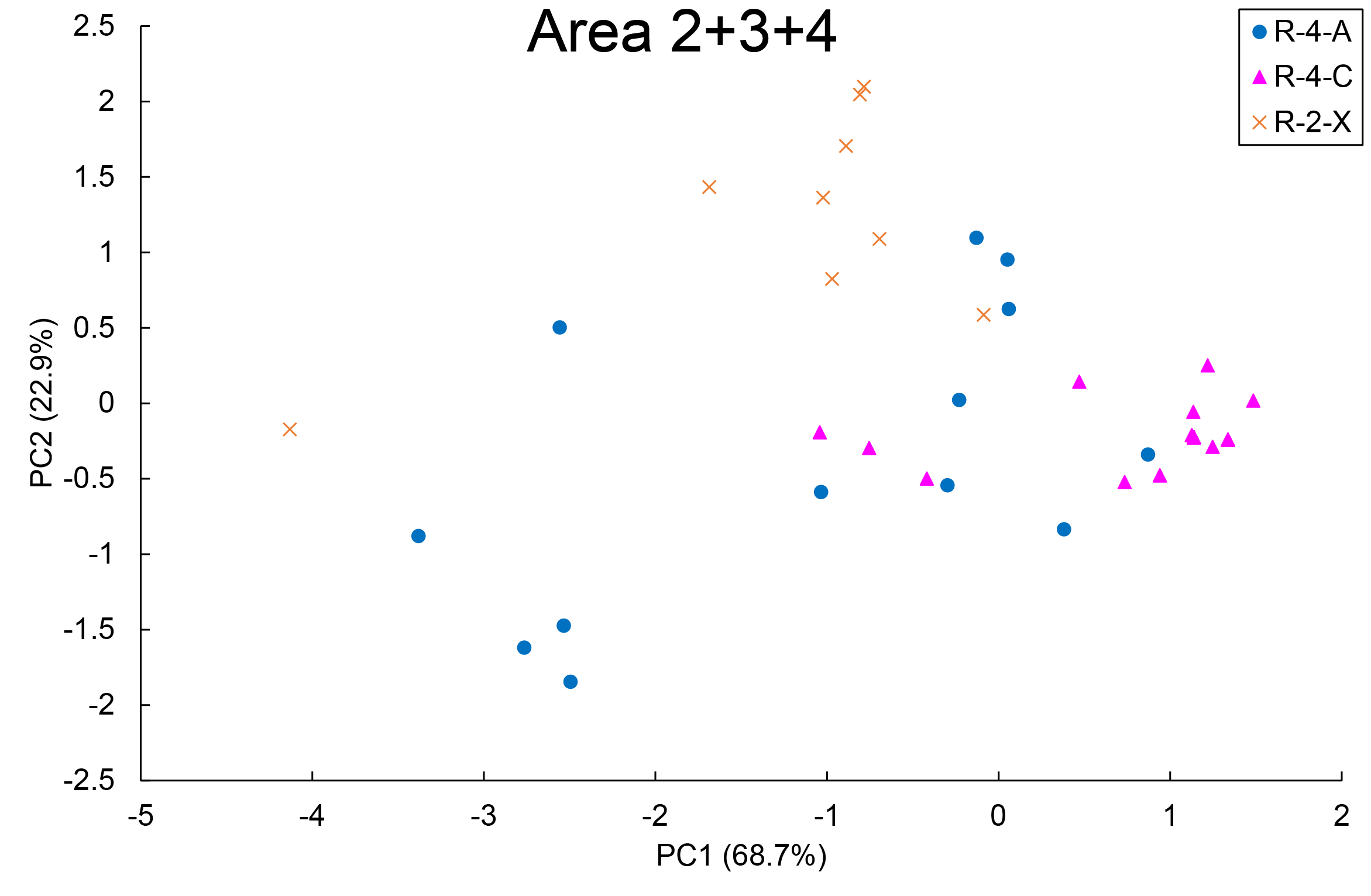

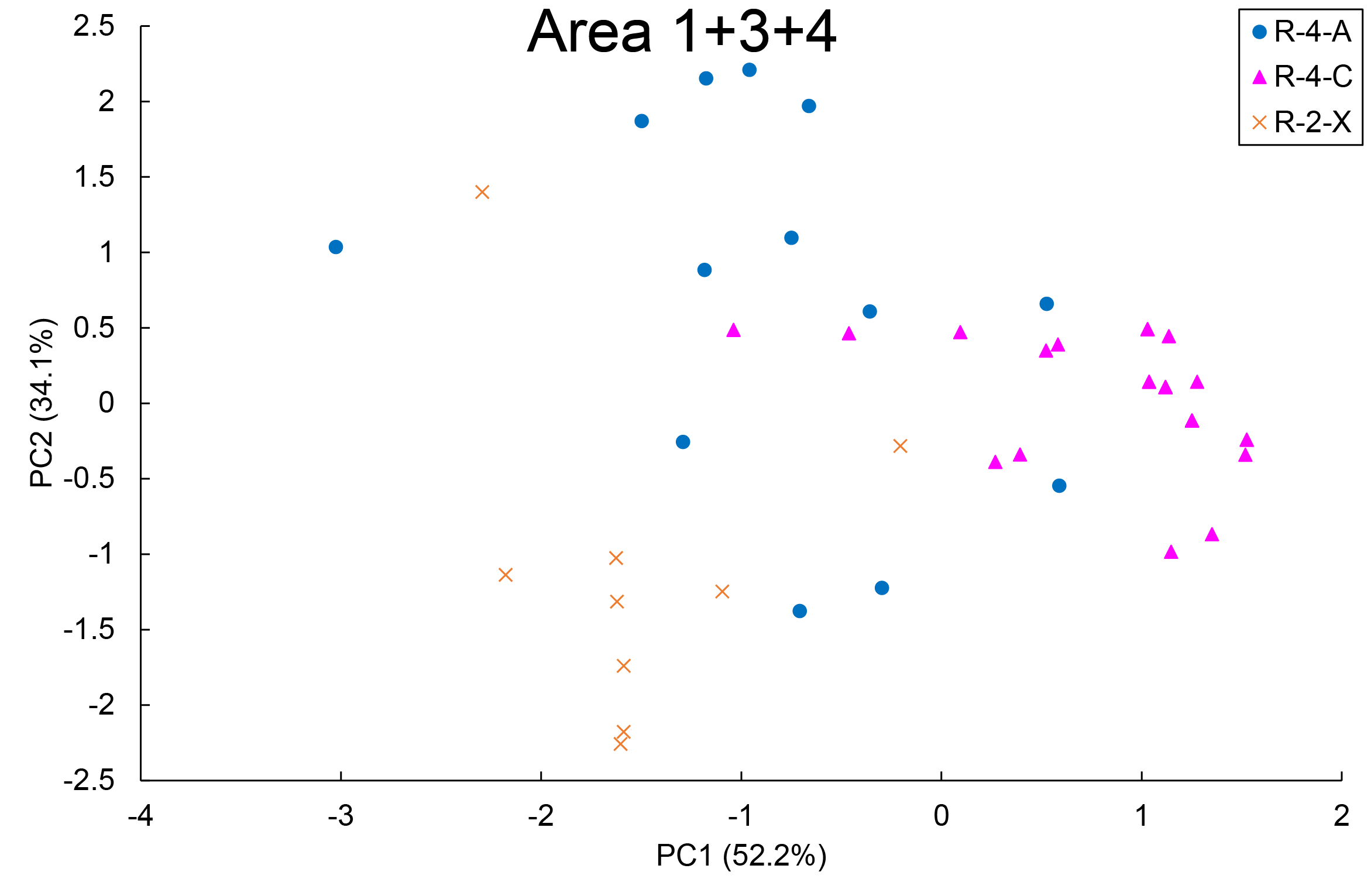


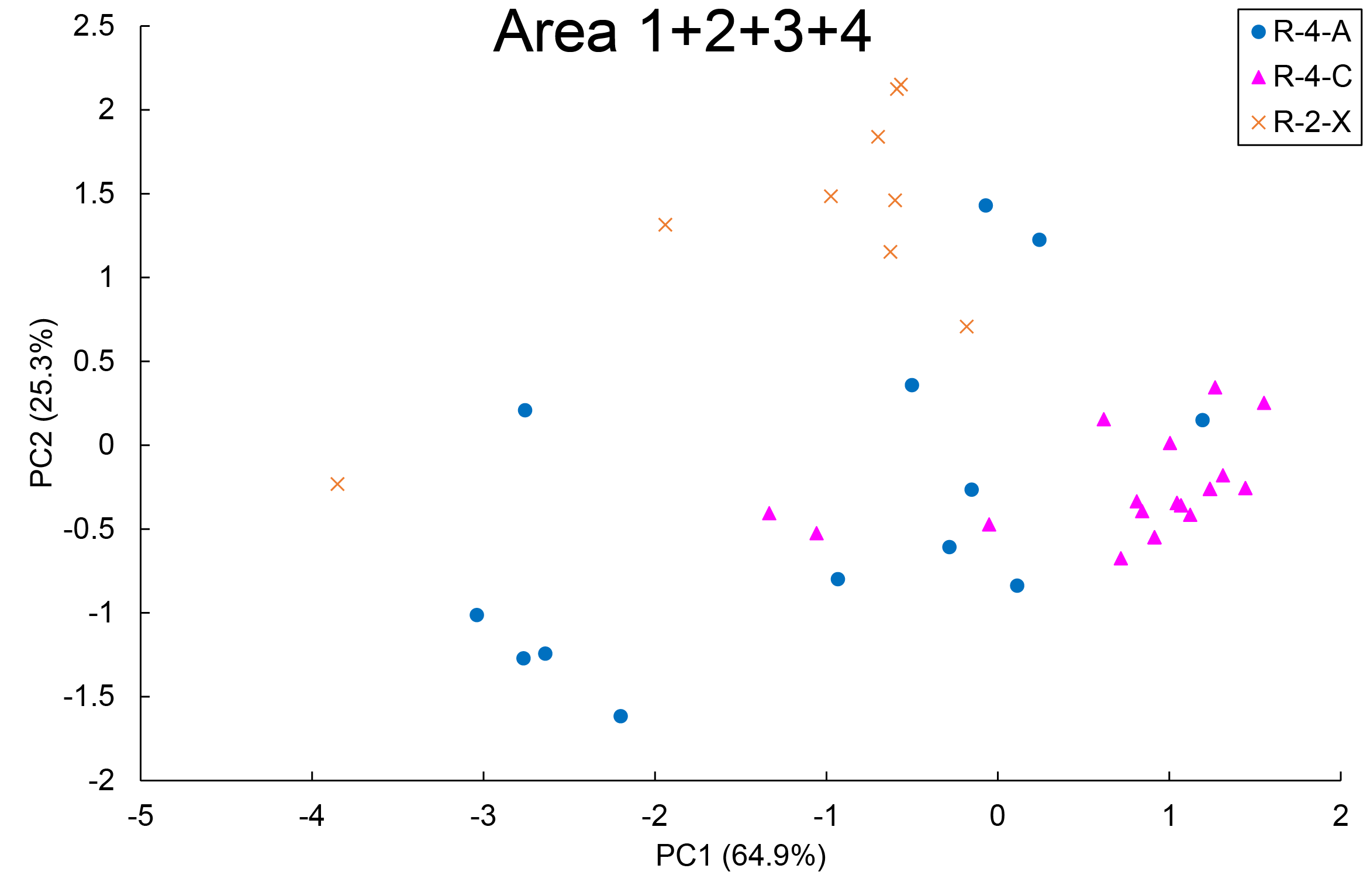

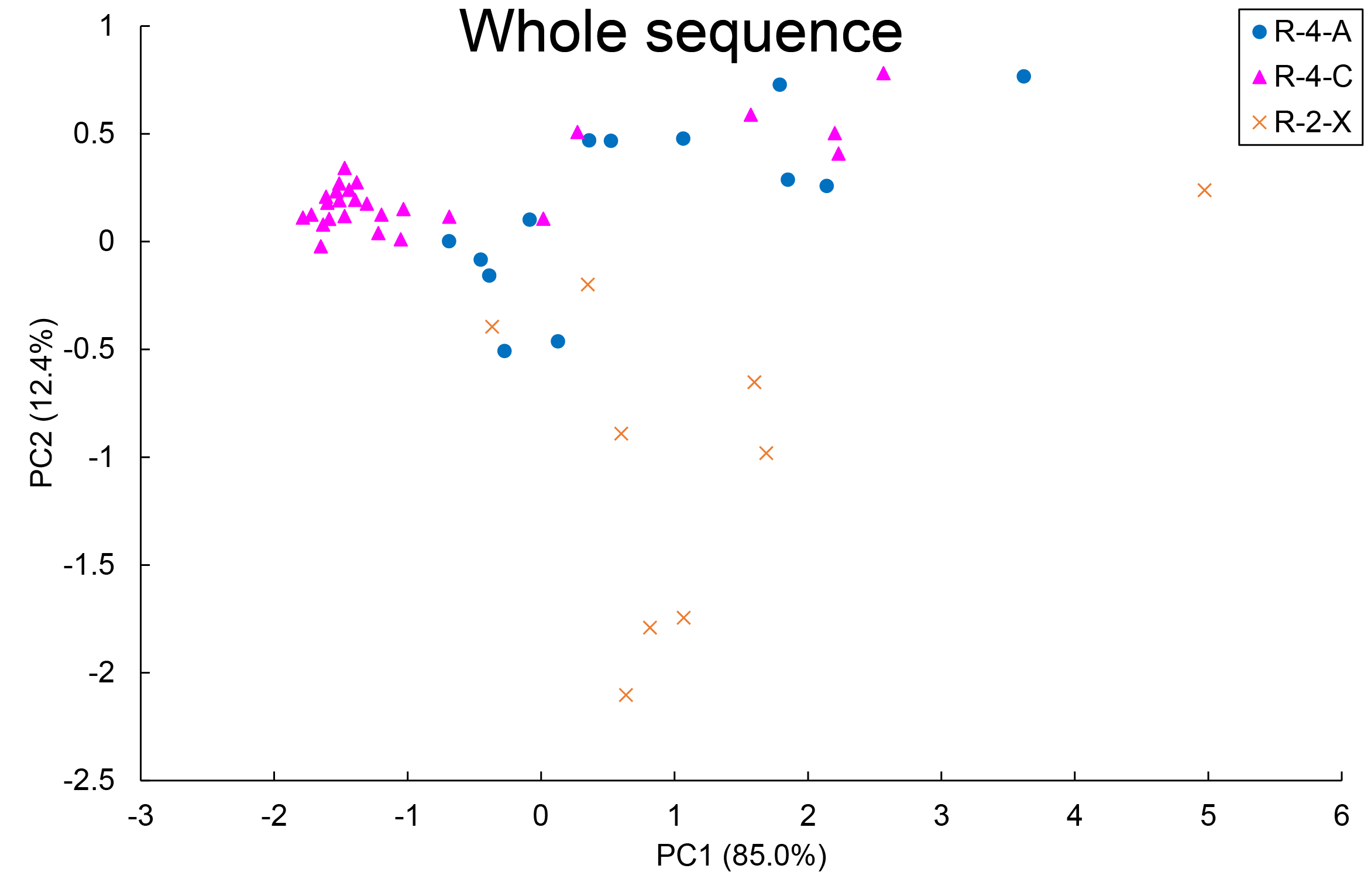
Fig. S2. PCA on the three HMM scores. The results are shown for each combination of Areas used in pHMM construction. The percentage in parentheses indicates the proportion of variance for the component.


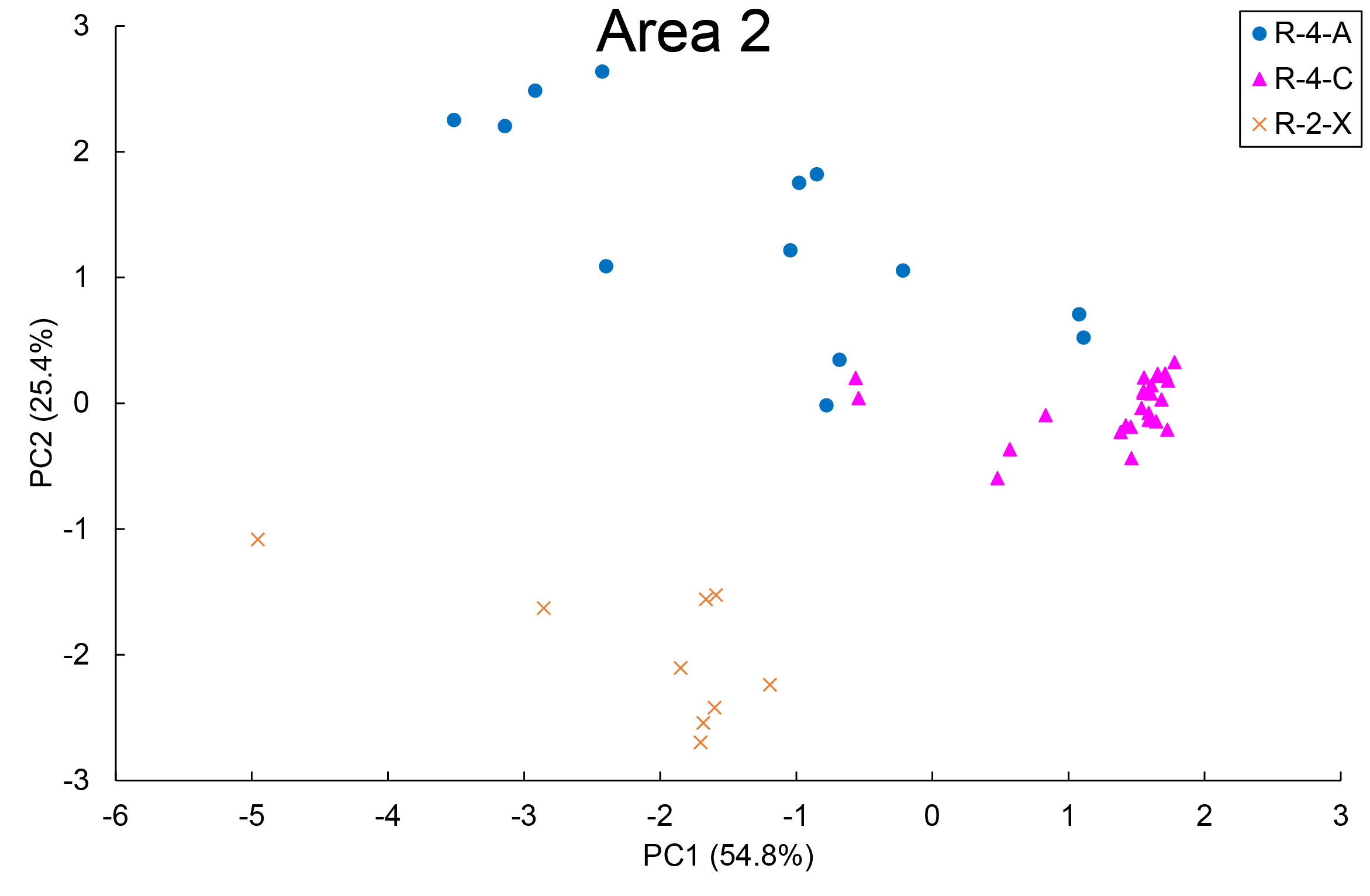

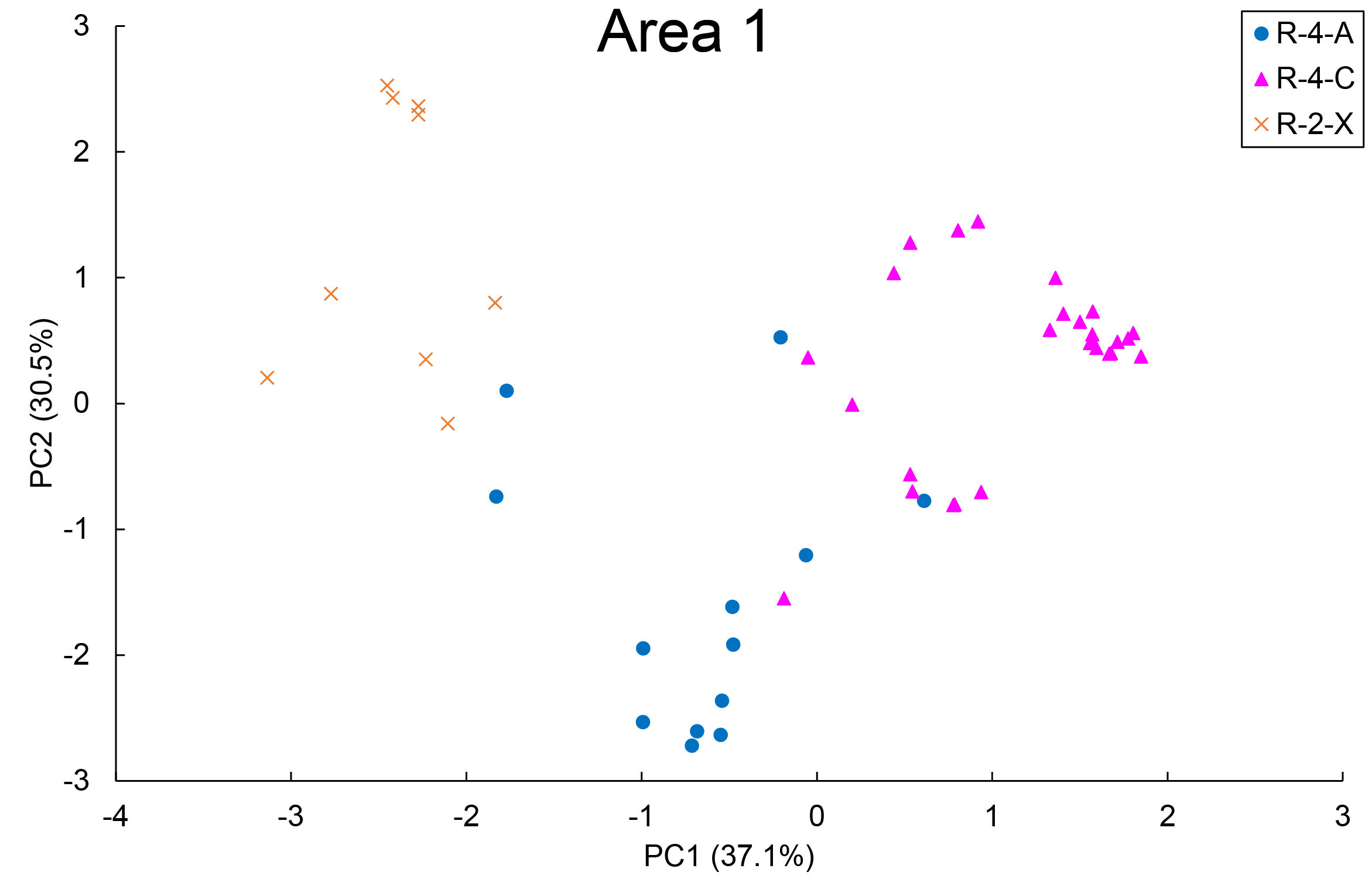


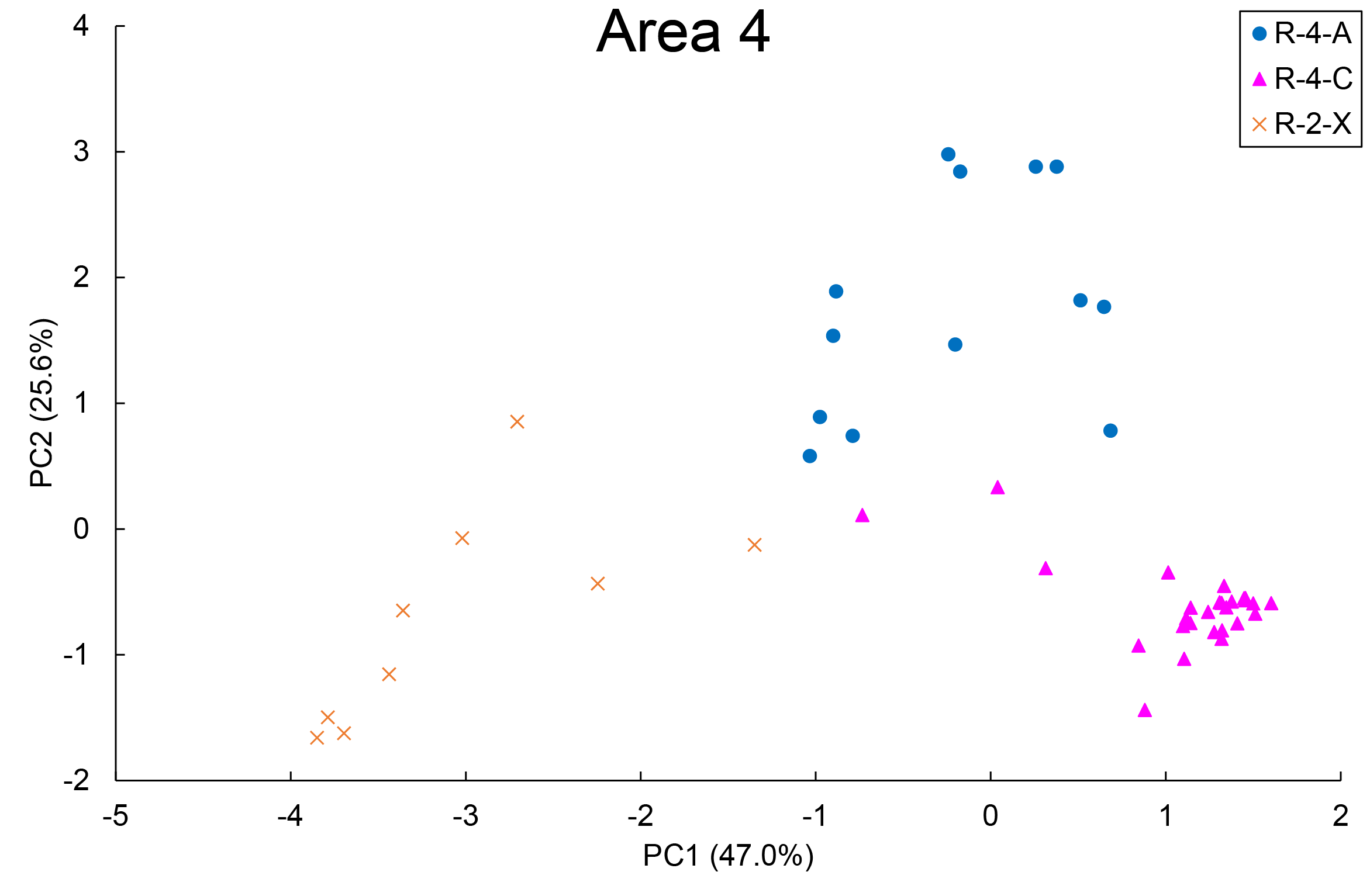

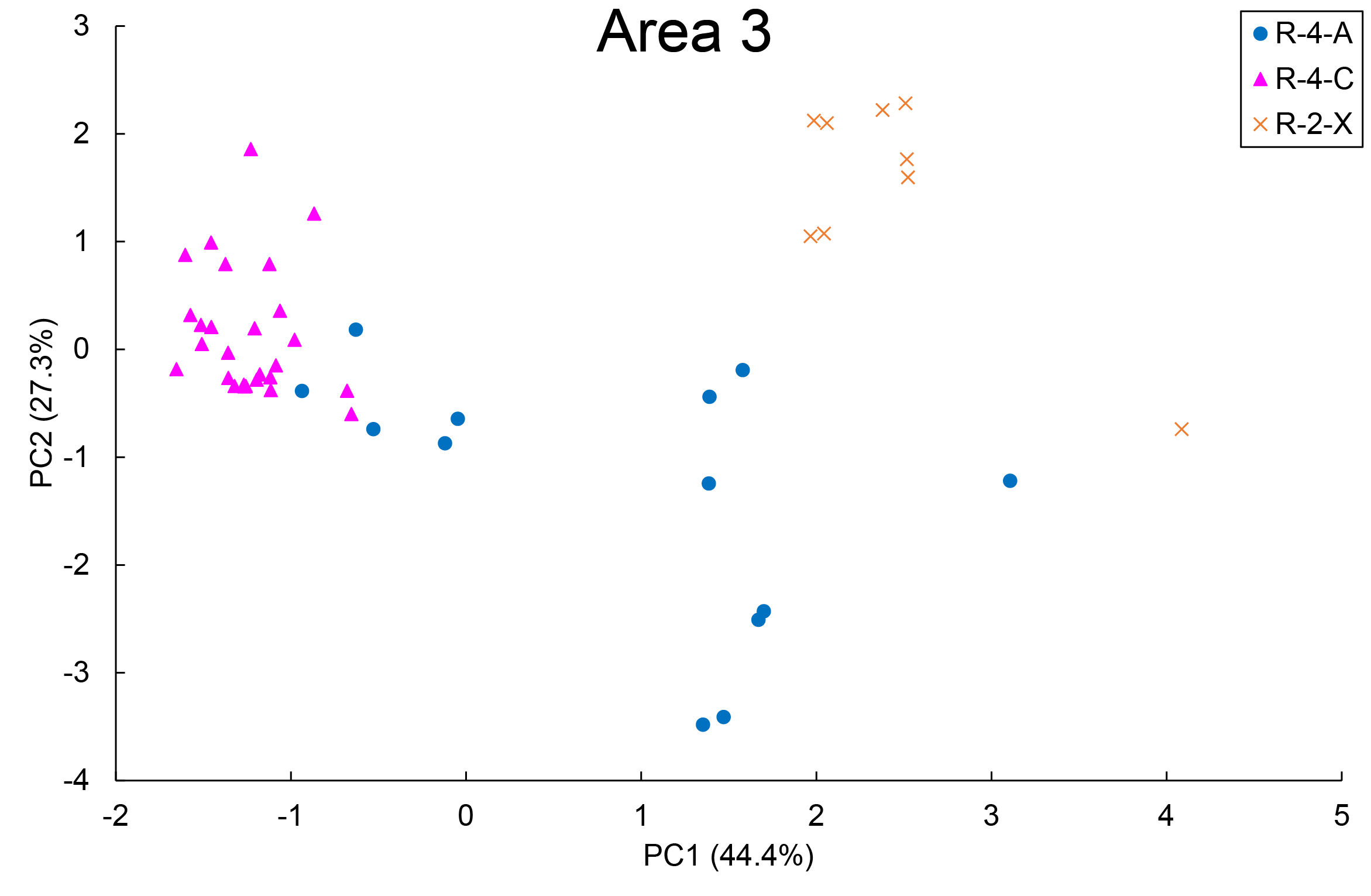


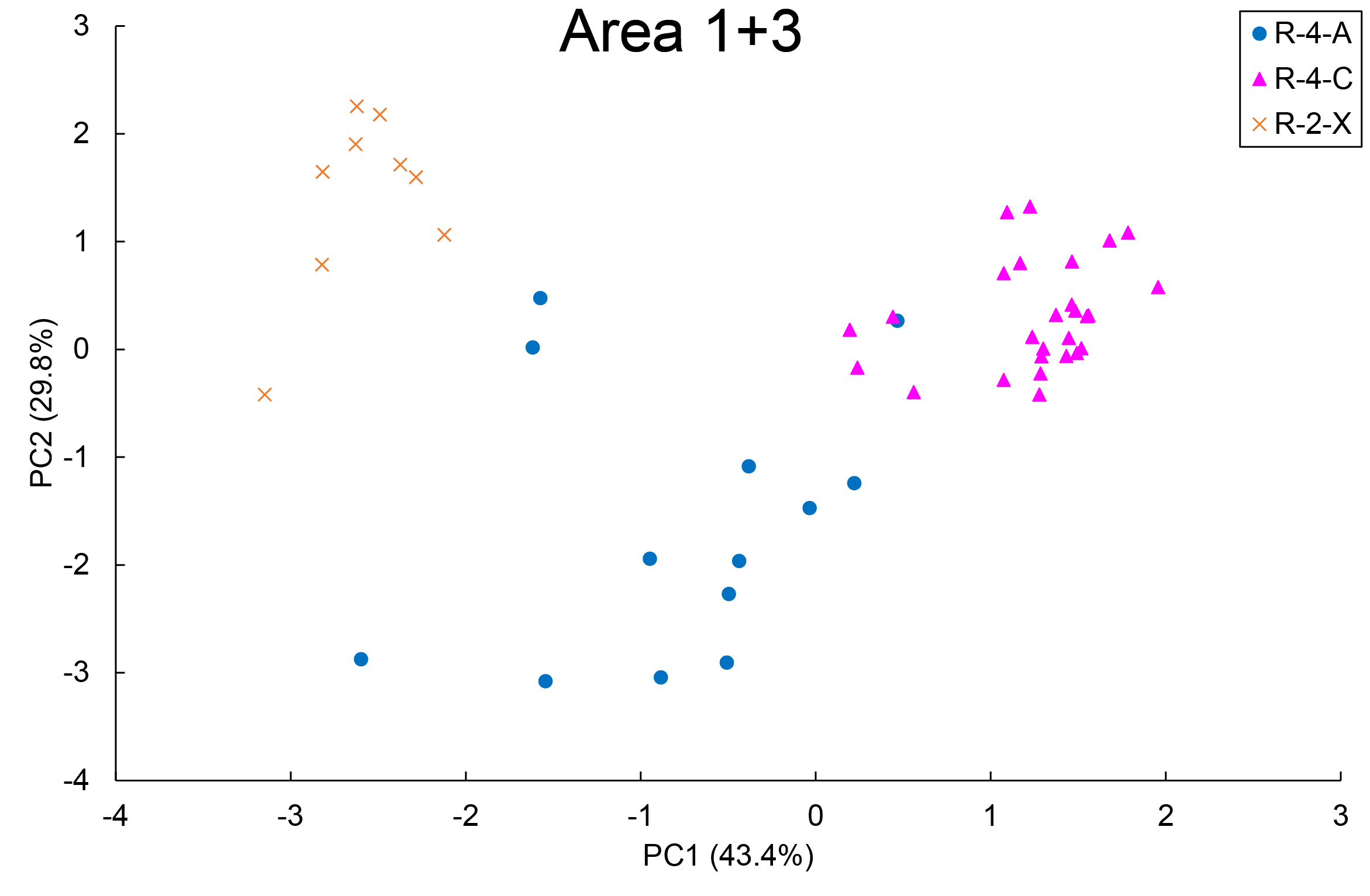

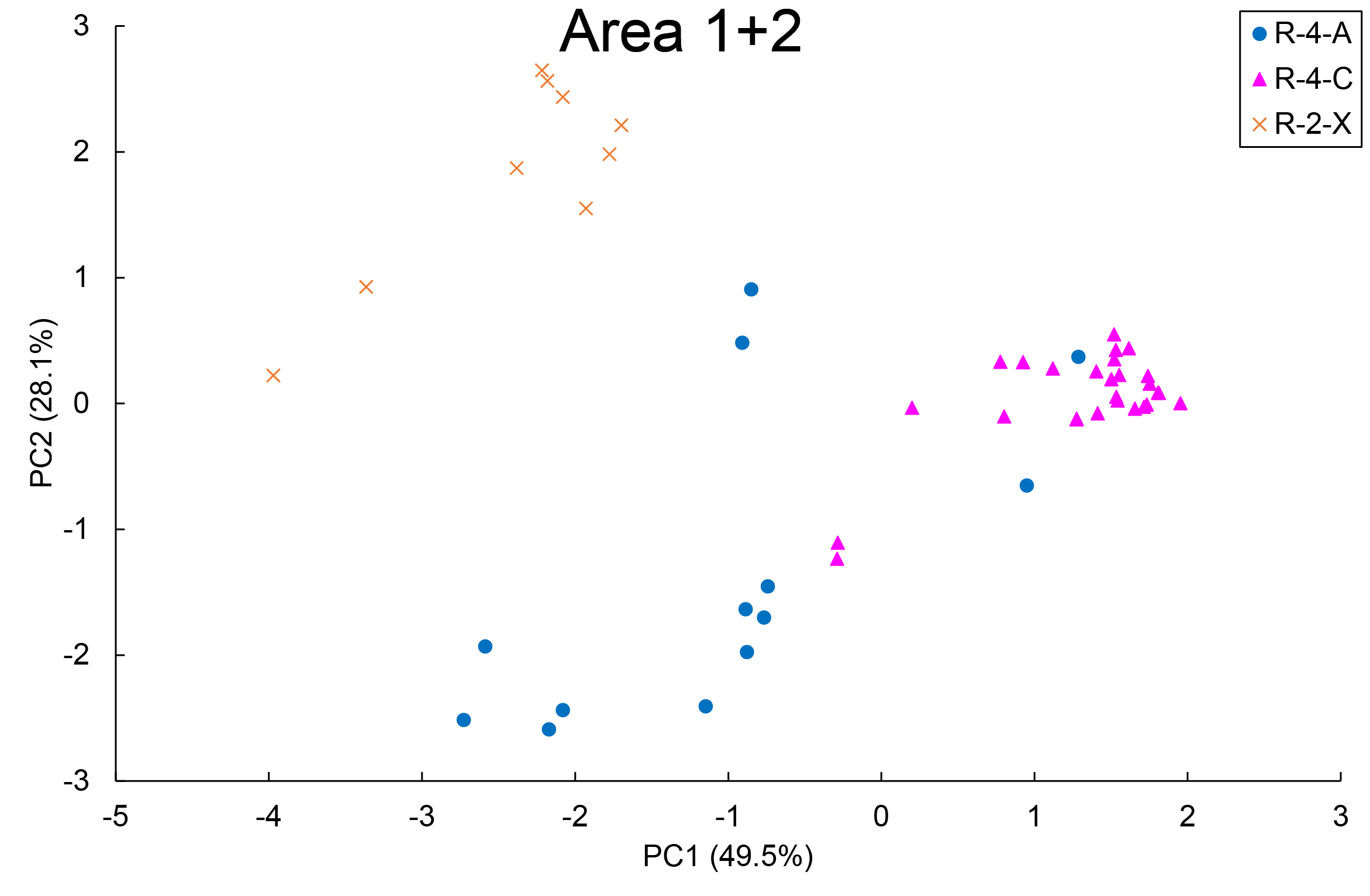


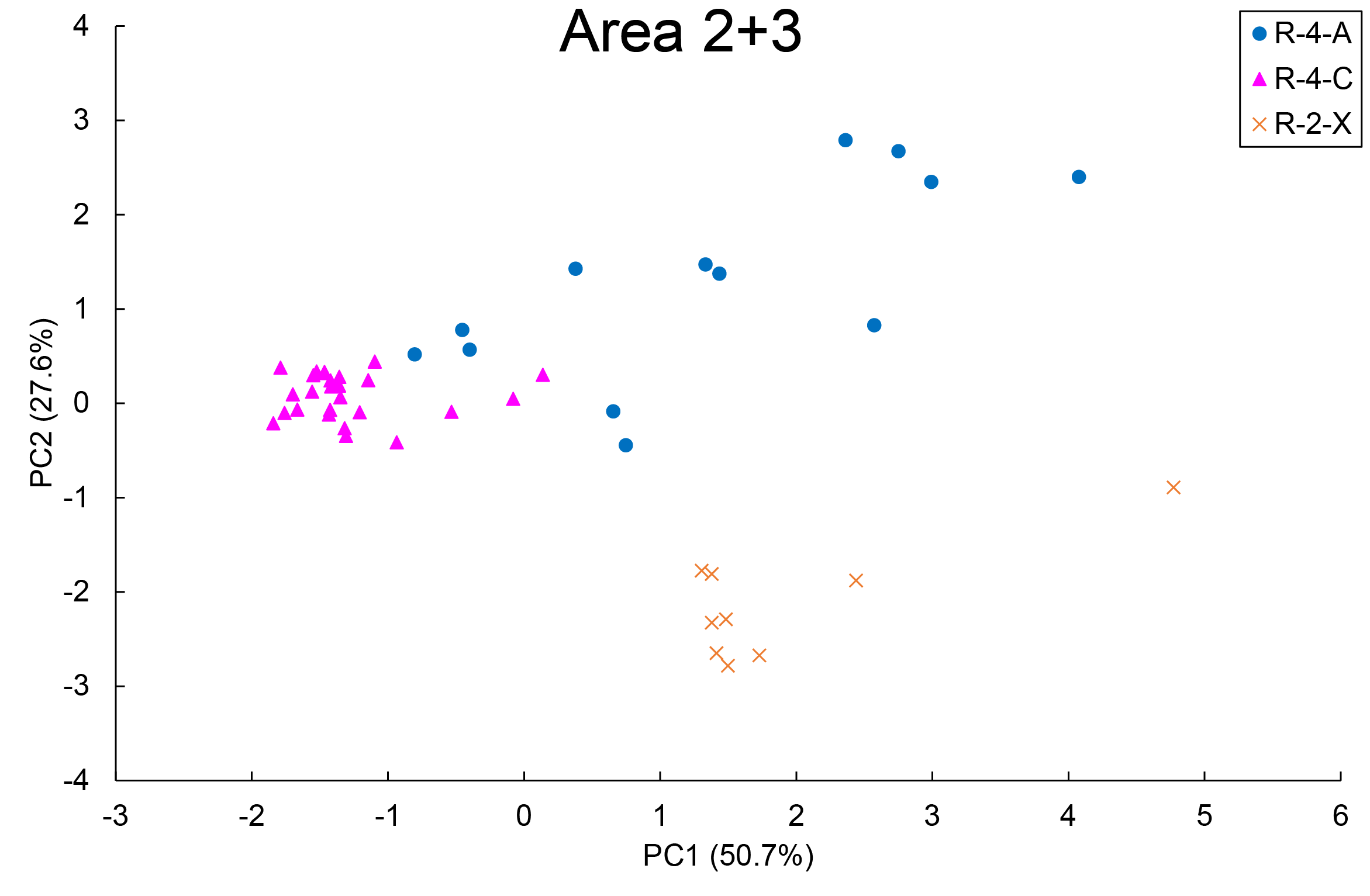

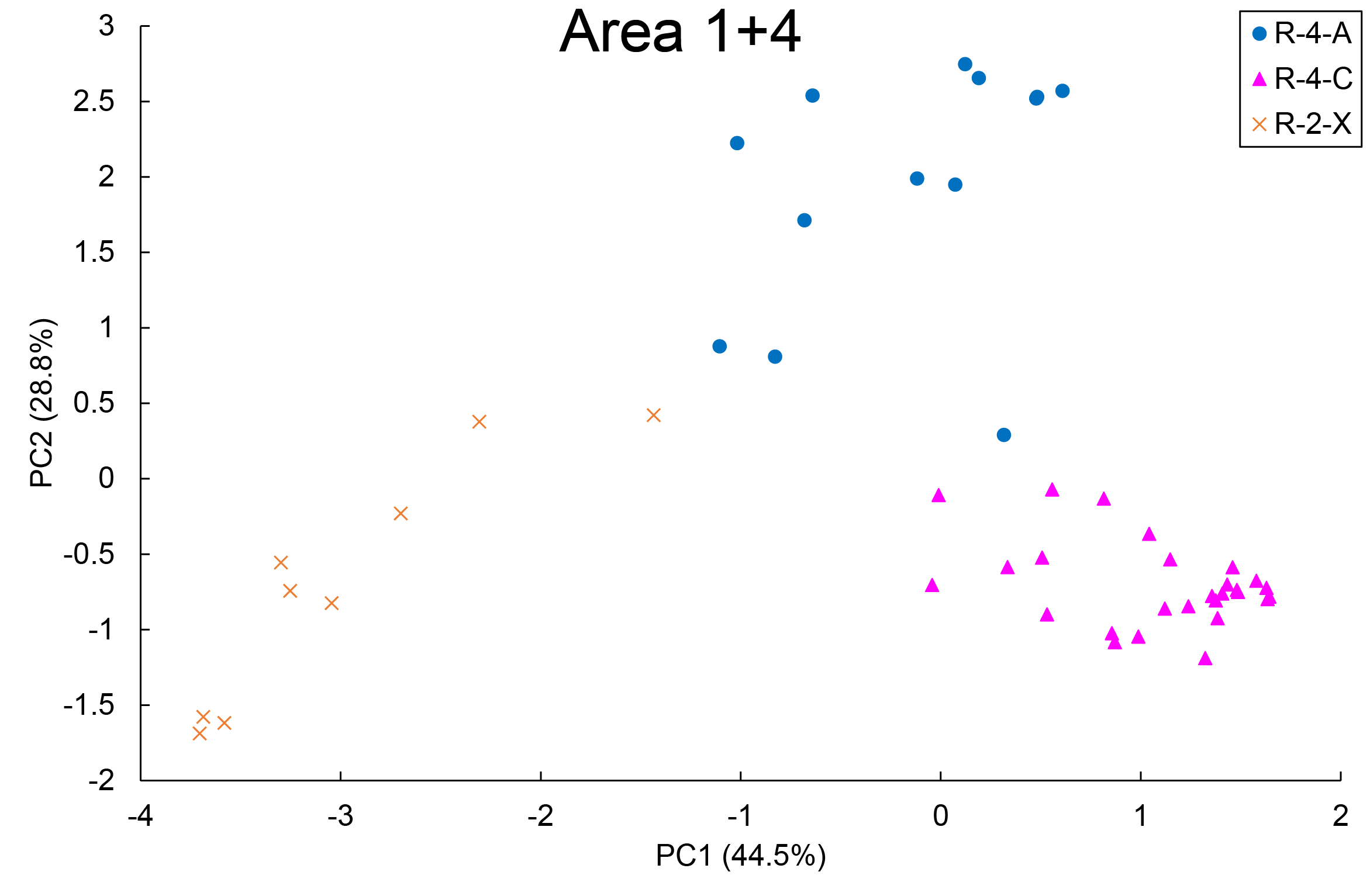


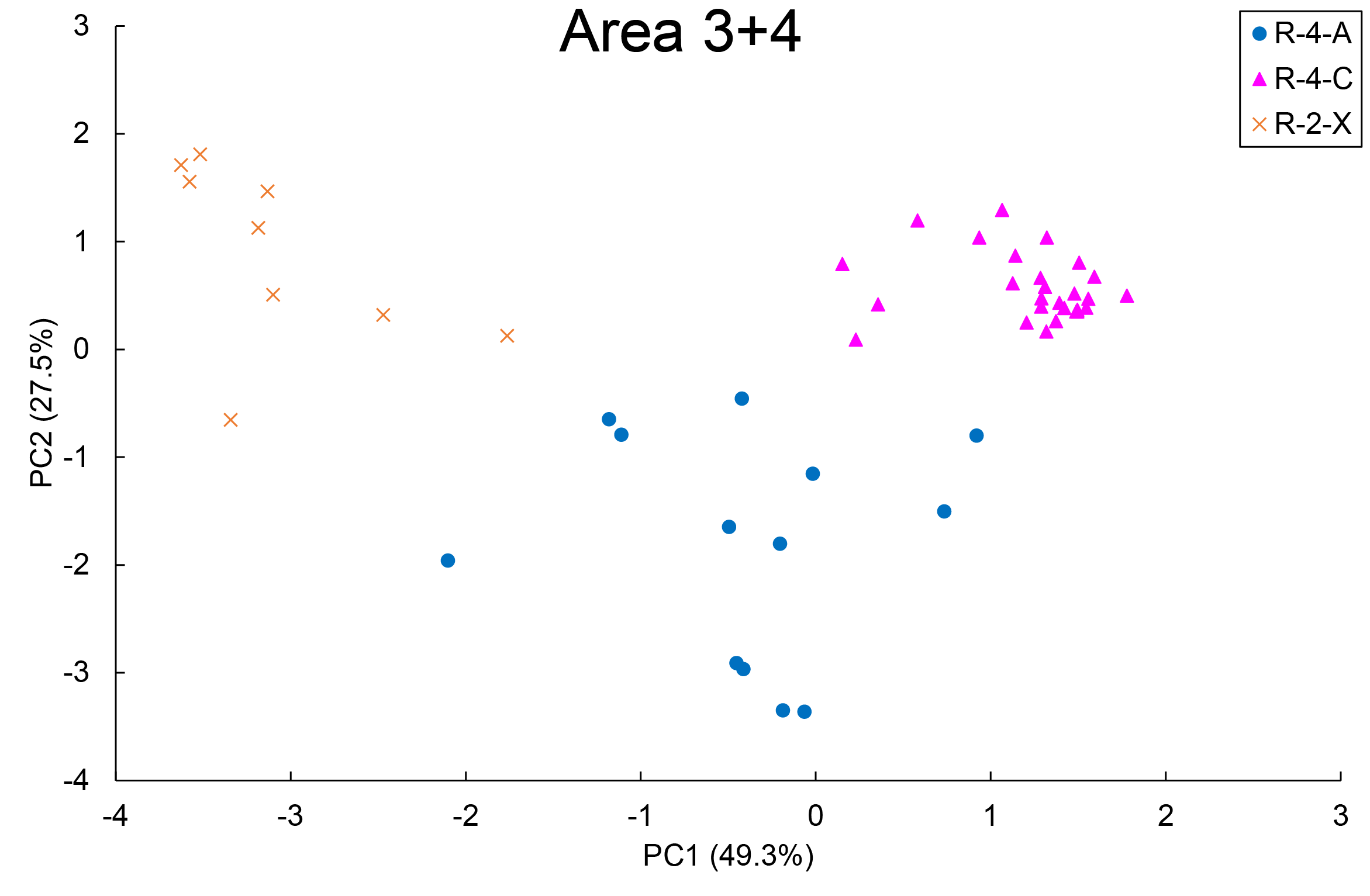

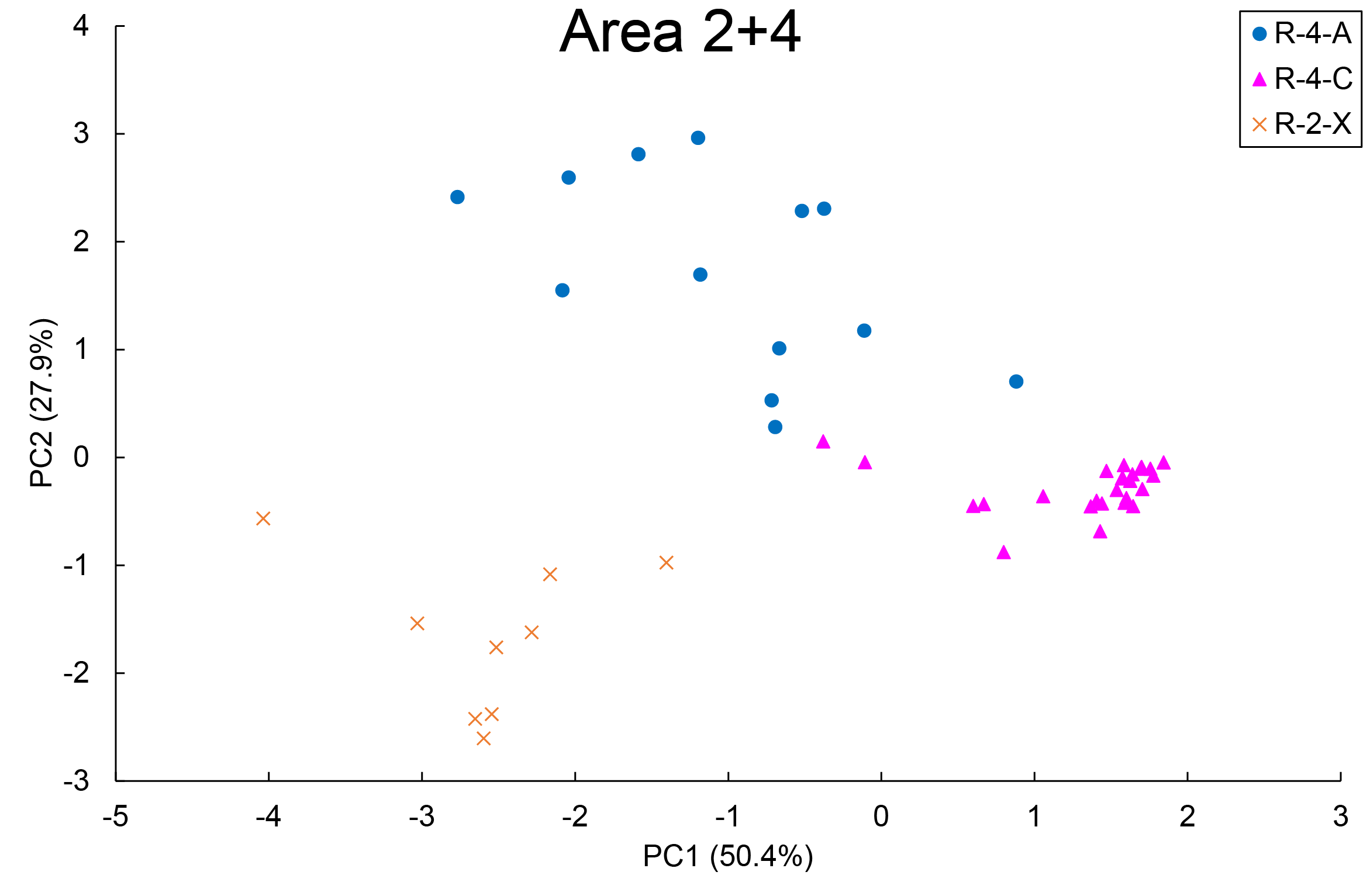


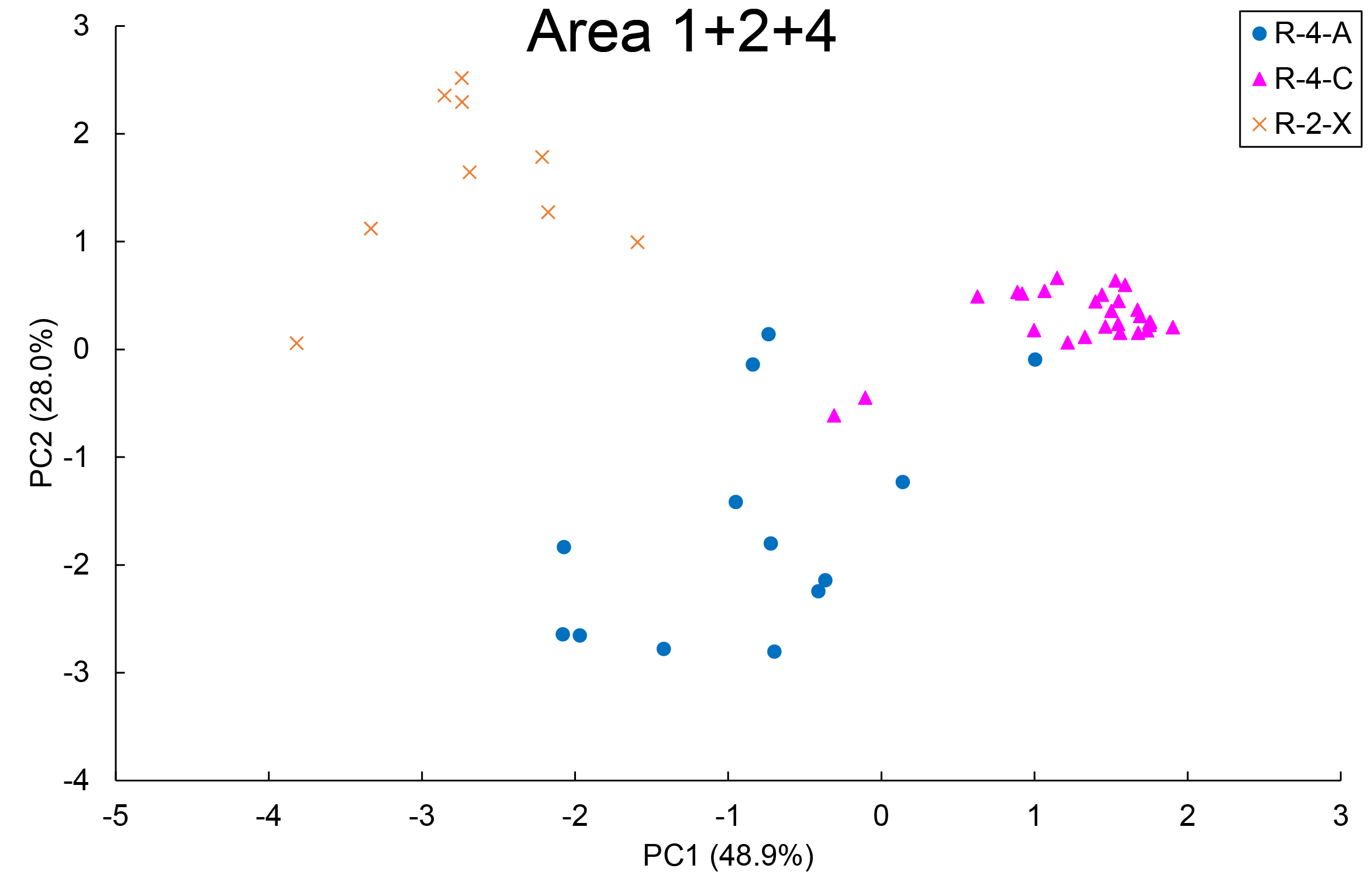

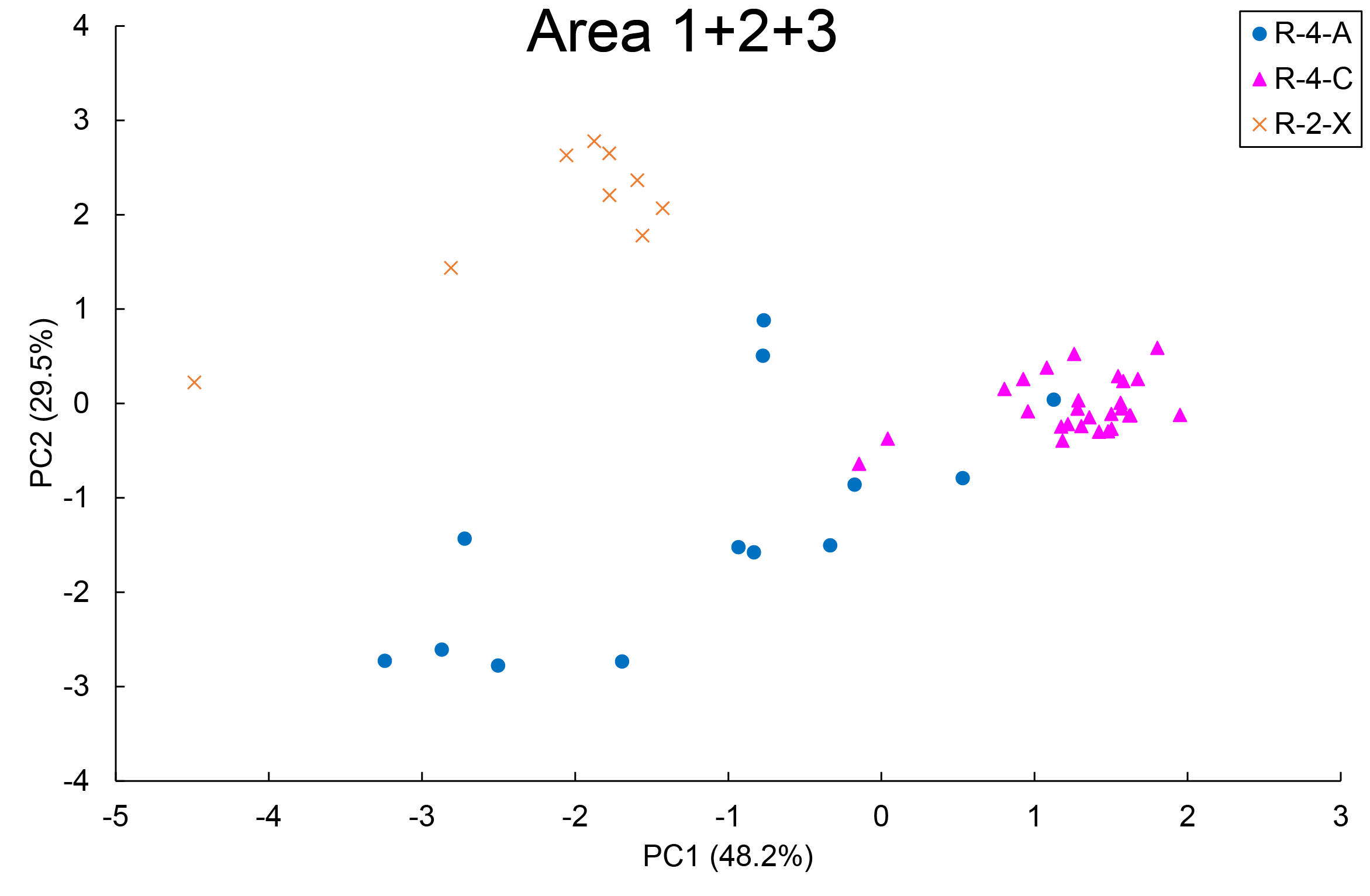


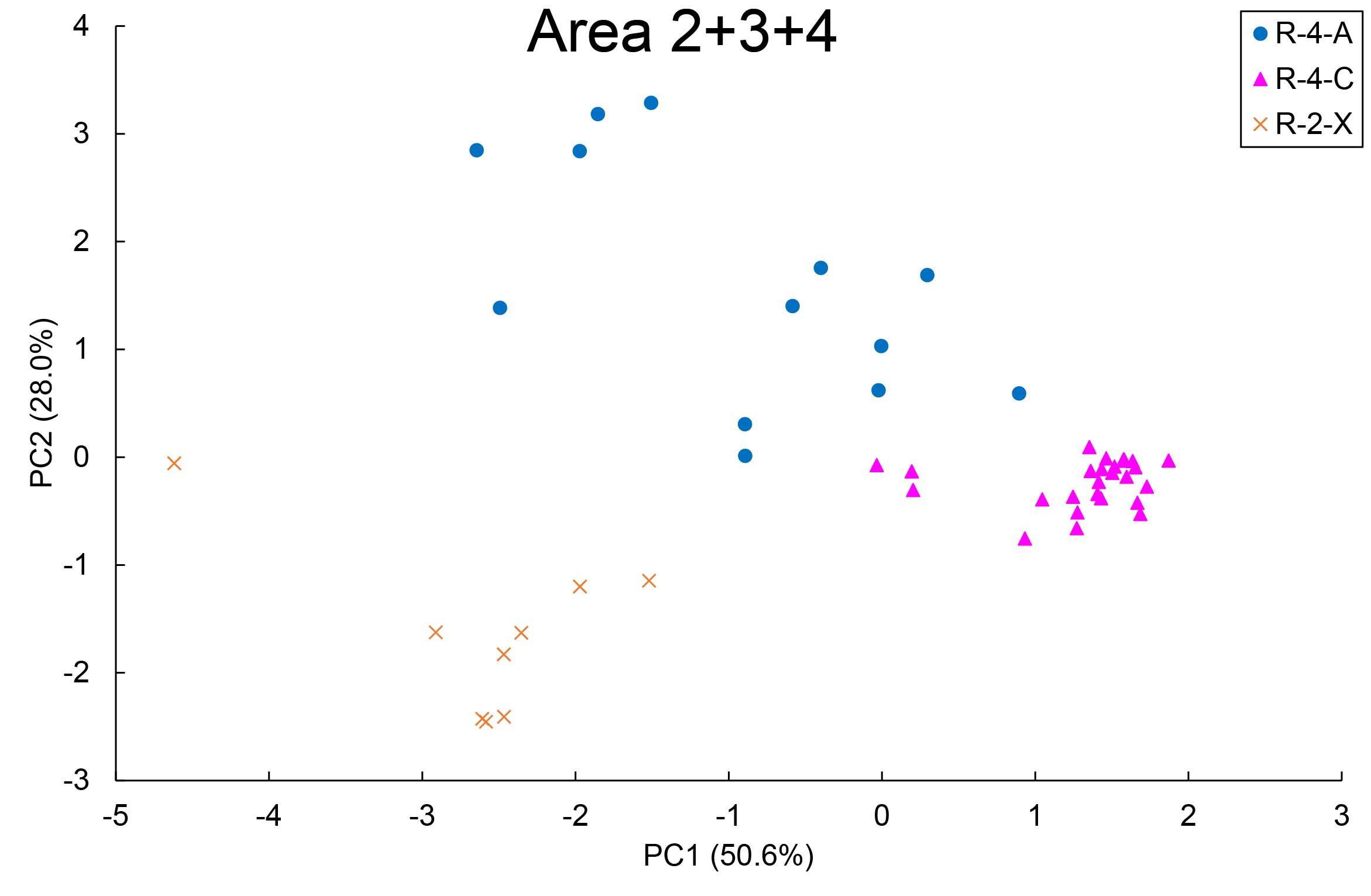

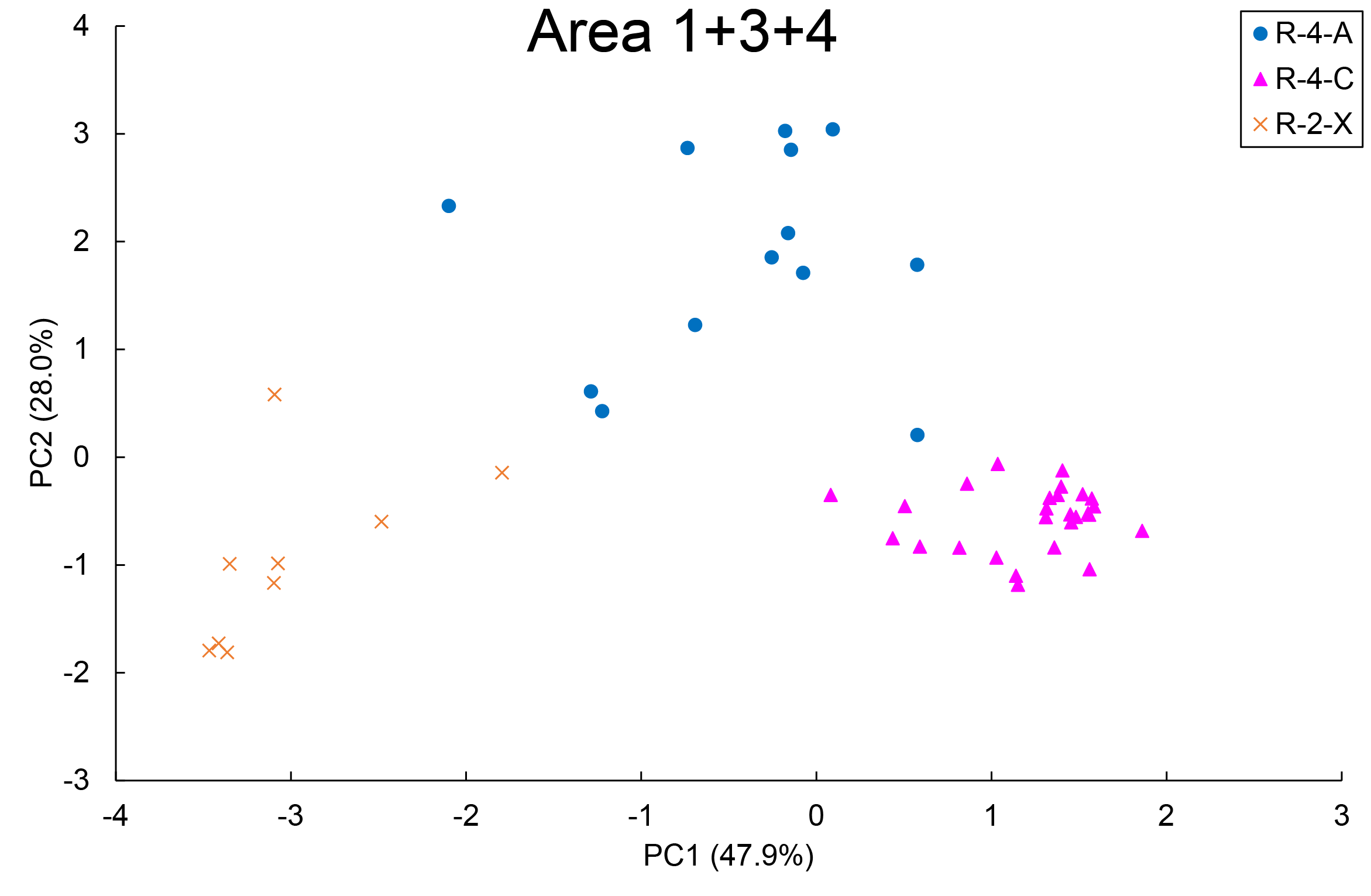


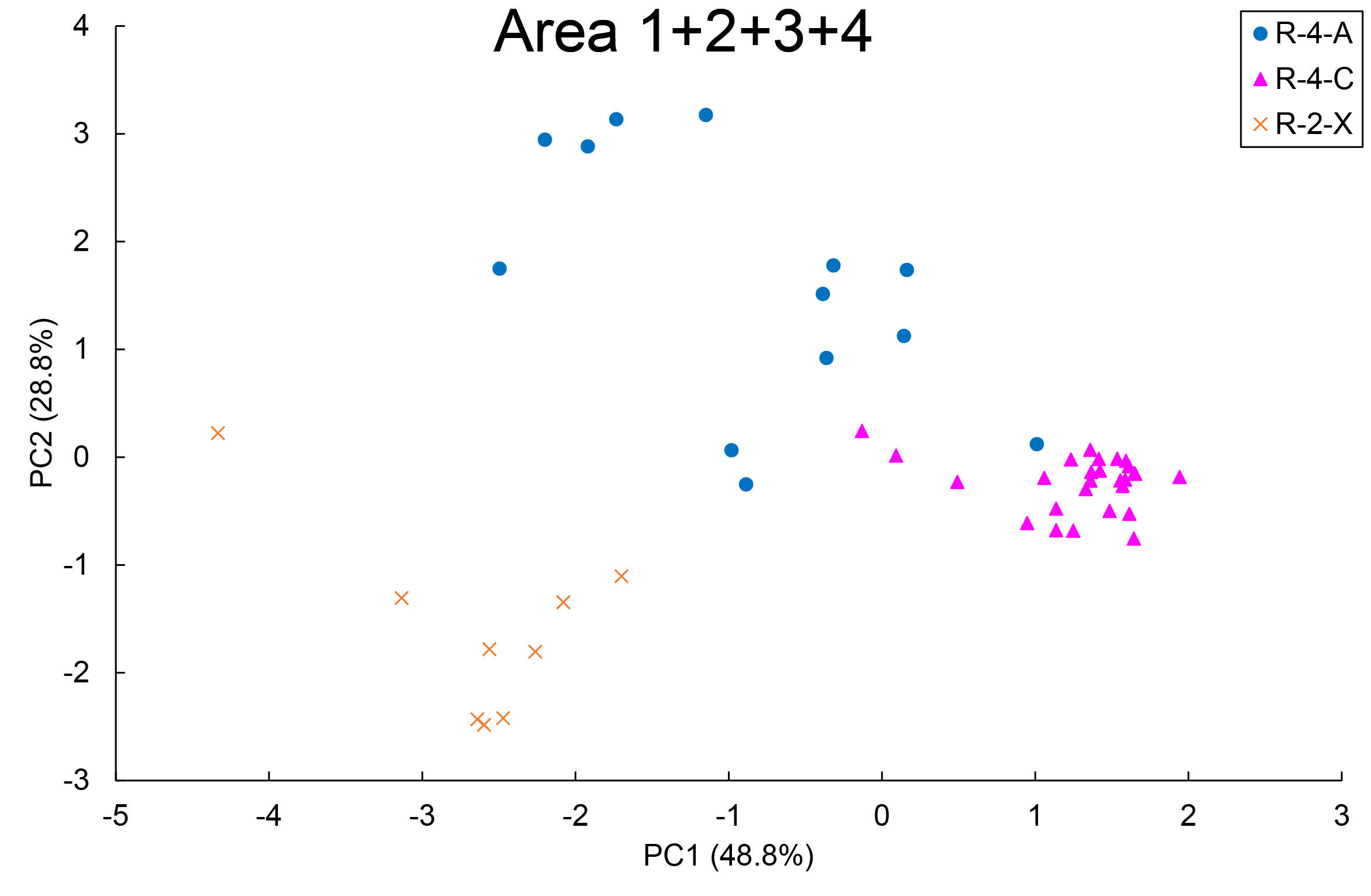

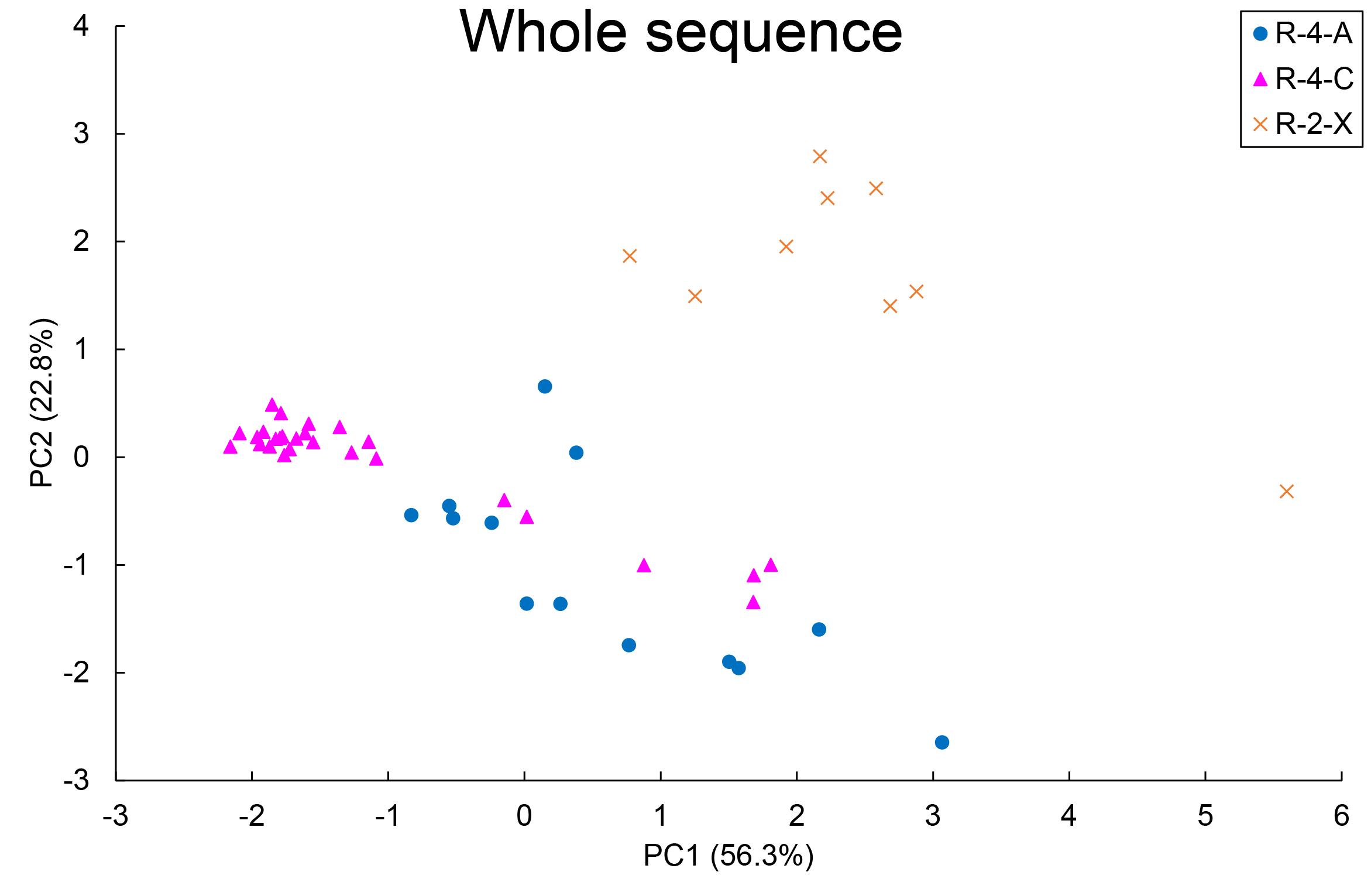
Fig. S3. Results of PCA on the six scores (three HMM and three correlation scores) for each combination of Areas used in pHMM construction. The percentage in parentheses indicates the proportion of variance for the component.

Fig. S4. Phylogenetic tree of ketosynthase domains of plant type III PKSs extracted from complete genomes in KEGG GENES. The predicted reaction types of the PKSs are indicated by the colors: magenta, R-4-C; light blue, R-4-A; orange, R-2-X; gray, other. The reaction types of PKSs with known reactions are indicated by the colors: red, R-4-C; blue, R-4-A; yellow, R-2-X; black, other.
