# Supplementary material for: Discriminating the reaction types of plant type III polyketide synthases
Source: Bioinformatics. 2017 Mar 6;33(13):1937–43. doi: 10.1093/bioinformatics/btx112 (PMC5870536; doi:10.1093/bioinformatics/btx112)

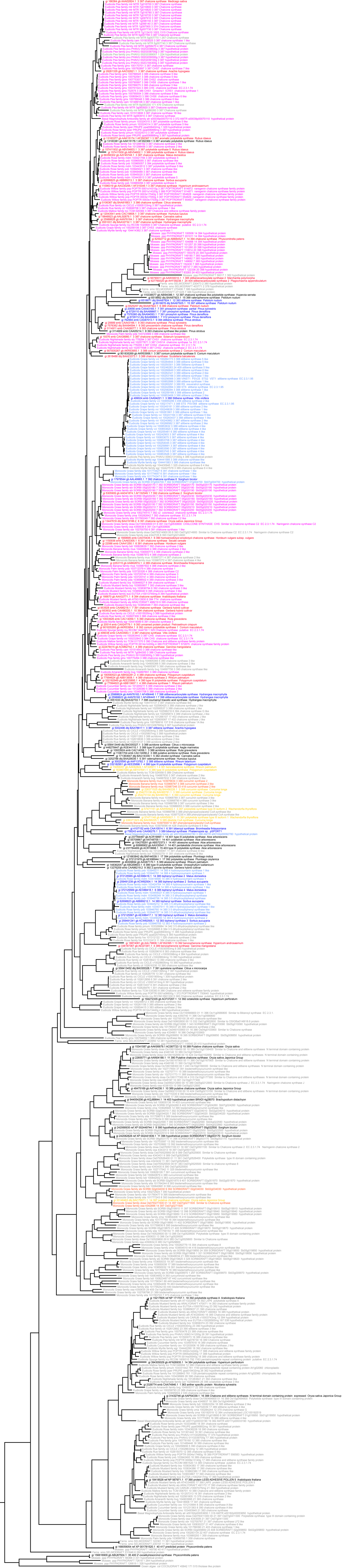

Supplement: Supplementary Data [file Supplementary Fig4.pdf › Supplementary Fig4.pdf]
